# New Cyclic Lipopeptides of the Iturin Class Produced by Saltern-Derived *Bacillus* sp. KCB14S006

**DOI:** 10.3390/md14040072

**Published:** 2016-04-02

**Authors:** Sangkeun Son, Sung-Kyun Ko, Mina Jang, Jong Won Kim, Gil Soo Kim, Jae Kyoung Lee, Eun Soo Jeon, Yushi Futamura, In-Ja Ryoo, Jung-Sook Lee, Hyuncheol Oh, Young-Soo Hong, Bo Yeon Kim, Shunji Takahashi, Hiroyuki Osada, Jae-Hyuk Jang, Jong Seog Ahn

**Affiliations:** 1 Anticancer Agent Research Center, Korea Research Institute of Bioscience and Biotechnology, Cheongju 363-883, Korea; sonski@kribb.re.kr (S.S.); ksk1230@kribb.re.kr (S.-K.K.); jangmina@kribb.re.kr (M.J.); jwkim@kribb.re.kr (J.W.K.); sssktu@kribb.re.kr (G.S.K.); scarscar@kribb.re.kr (J.K.L.); eunsooj@kribb.re.kr (E.S.J.); ijryoo@kribb.re.kr (I.-J.R.); hongsoo@kribb.re.kr (Y.-S.H.); bykim@kribb.re.kr (B.Y.K.); 2 University of Science and Technology (UST), Daejeon 305-333, Korea; 3 Chemical Biology Research Group, RIKEN Center for Sustainable Research Science, Saitama 351-0198, Japan; futamuray@riken.jp (Y.F.); shunjitaka@riken.jp (S.T.); hisyo@riken.jp (H.O.); 4 Korean Collection for Type Cultures, Korea Research Institute of Bioscience and Biotechnology, Jeongeup 56212, Korea; jslee@kribb.re.kr; 5 College of Pharmacy, Wonkwang University, Iksan 570-749, Korea; hoh@wonkwang.ac.kr; 6 RIKEN-KRIBB Joint Research Unit, RIKRN Global Research Cluster, Saitama 351-0198, Japan

**Keywords:** saltern-derived bacteria, iturins, antifungal, cytotoxic, indoleamine-2,3-dioxygenase

## Abstract

Salterns, one of the most extreme natural hypersaline environments, are a rich source of halophilic and halotolerant microorganisms, but they remain largely underexplored ecological niches in the discovery of bioactive secondary metabolites. In continued efforts to investigate the metabolic potential of microbial populations from chemically underexplored sites, three new lipopeptides named iturin F_1_, iturin F_2_ and iturin A_9_ (**1**–**3**), along with iturin A_8_ (**4**), were isolated from *Bacillus* sp. KCB14S006 derived from a saltern. The structures of the isolated compounds were established by 1D-, 2D-NMR and HR-ESIMS, and their absolute configurations were determined by applying advanced Marfey’s method and CD spectroscopy. All isolates exhibited significant antifungal activities against various pathogenic fungi and moderate cytotoxic activities toward HeLa and *src*^ts^-NRK cell lines. Moreover, in an *in vitro* enzymatic assay, compound **4** showed a significant inhibitory activity against indoleamine 2,3-dioxygenase.

## 1. Introduction

*Bacillus* lipopeptides, represented by three classes, namely the iturins, surfactins and fengycins, have been widely studied for their effective antibacterial or antifungal activities [1]. Among them, iturins, which possess a heptapeptide backbone connected to a C_13_ to C_17_ β-amino fatty acid chain, exhibit strong *in vitro* fungitoxicity through the formation of ion-conducting pores on fungal membranes [1,2]. They exhibit structural heterogeneity at the amino acid residues as well as in their length and branching of the fatty acid chain. Some classic examples of these amphiphilic compounds include iturins A, C, D and E, bacillomycins D, F and L, bacillopeptin and mycosubtilin, all of which are arranged in an lddlldl configurational sequence [3]. Especially, iturin A is composed of up to eight isomers (iturin A_1_–A_8_) with different lengths (10–14 carbons) and branching (*n*-, *iso*- or *anteiso*-configurations) of the fatty acid chain [4].

The exploration of biologically active secondary metabolites has provided a number of small molecules extremely useful as molecular probes and drug leads. However, the high probability of rediscovering known compounds with a conventional approach using readily accessible soil microorganisms emphasized the importance of studying untapped resources for novel chemistry [5]. Halophilic and halotolerant bacteria isolated from saline environments, such as oceans and saline lakes, have attracted the attention of natural product chemists [6,7]. It is primarily due to their unique adaptive strategies which could lead them to produce novel metabolites. Although salterns are a popular environment for studies on the physiology of halophilic and halotolerant microorganisms, they are also one of the least explored habitats for novel secondary metabolites. Several recent studies have revealed that microorganisms from salterns, mainly actinobacteria and fungi, produce various secondary metabolites with interesting bioactivities, and supported their metabolic potential [8,9,10]. In an effort to discover new metabolites with pharmaceutical potential from halophilic and halotolerant bacteria, we performed chemical screening of extracts obtained from saltern-derived bacteria using a dereplication strategy based on public and in-house databases [11,12]. This investigation, followed by scale-up culture, solvent extraction and reversed-phase chromatography, resulted in the isolation of three new lipopeptides (**1**–**3**), together with known compound iturin A_8_ (**4**), from *Bacillus* sp. KCB14S006. Compounds **1** and **2** which incorporate 4-OH-Pro in the peptide backbone are considered the first members of the iturin class containing a modified amino acid. All the isolates were tested for their antimicrobial and cytotoxic activities, as well as their enzyme inhibition activities. We describe herein the bacterial cultivation, isolation, structural elucidation and biological activities of compounds **1**–**4**.

## 2. Results

### 2.1. Isolation of Compounds

**Figure 1:**
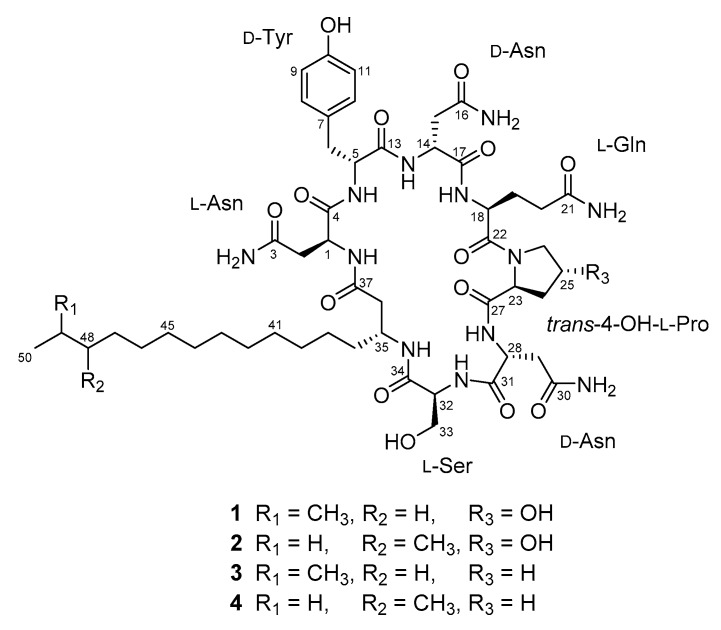
The structures of compounds **1**–**4** from *Bacillus* sp. KCB14S006.

The strain KCB14S006 was isolated from a saltern in Incheon, Korea, and was characterized as belonging to the genus Bacillus by phylogenetic analysis based on 16S rRNA gene sequences. It was maintained on marine agar at 28 °C, and cultured in square petri dishes containing the same medium for the large-scale culture. Seven-day-old cultures were extracted with acetone and concentrates were partitioned between EtOAc-H_2_O. The EtOAc soluble portion was further separated by reversed-phase chromatography to yield four pure compounds **1**–**4** (Figure 1).

### 2.2. Structure Determination

**Figure 2:**
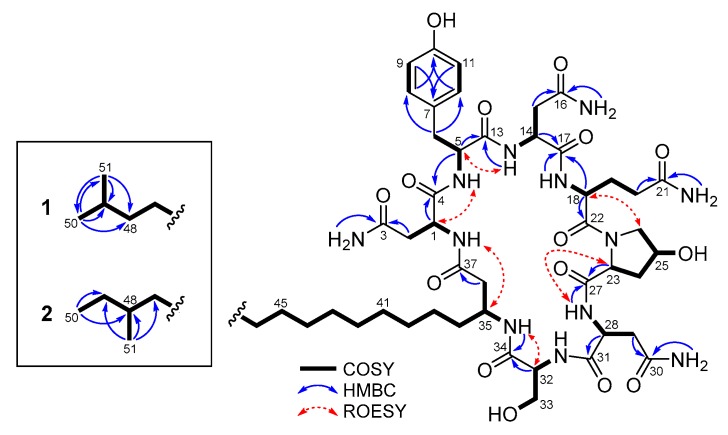
Key 2D NMR correlations of **1** and terminal groups of **2**.

Iturin F_1_ (**1**) was isolated as a white amorphous powder. Its molecular formula was determined to be C_51_H_80_N_12_O_15_ on the basis of HR-ESIMS in combination with the ^1^H and ^13^C NMR spectroscopic data (Table 1). The characteristic signals in the ^1^H and ^13^C NMR spectra of **1** in DMSO-*d*_6_, including α-methine protons, amide carbonyls and a series of overlapping protons at 1.24 ppm, indicated the lipopeptidic nature of the compound. The measurement of the HSQC-DEPT spectrum revealed 18 exchangeable protons, one para-substituted benzene (δ_C/H_ 129.8/7.03 and 115.1/6.66), two geminal methyls (δ_C/H_ 22.5/0.84), one oxymethine (δ_C/H_ 68.7/4.39) and one oxymethylene (δ_C/H_ 61.4/3.69 and 3.65). Detailed analysis of the 1D and 2D NMR spectroscopic data, including DQF-COSY, TOCSY and HMBC, assigned overlapping sets of carbonyls, and constructed the individual residues as three Asn, one Tyr, one Gln, one 4-OH-Pro and one Ser (Figure 2). In particular, the presence of a 4-OH-Pro residue, which is new to the iturin class, was clearly shown by the chemical shift value of C-25 (δ_C_ 68.7). The structure of the β-amino fatty acid chain was determined mainly by the DQF-COSY and HMBC spectra, which was supported by its carbon chemical shifts. The ^1^H–^1^H sequence from equivalent geminal methyls H_3_-50 and H_3_-51 (δ_H_ 0.84) through the aliphatic methine H-49 (δ_H_ 1.49) to the long fatty acid chain, and the HMBC correlations of H_3_-50 and H_3_-51 with neighboring carbons, revealed an *iso*-type of the fatty acid chain in **1** [3]. Lastly, analysis of the HMBC and ROESY correlations between adjacent residues, together with consideration of the molecular formula and the unsaturation requirement, enabled us to establish the sequence of **1** as Asn_1_, Tyr, Asn_2_, Gln, 4-OH-Pro, Asn_3_, Ser and a β-amino fatty acid chain (Figure 2). Thus, the gross structure of **1** was established as shown in Figure 1. The heptapeptide backbones of the known iturin class consist of l and d forms of standard amino acids, except for methyl esters of Asp and Glu in iturin E which could be formed during the methanol extraction [13]. Therefore, the identification of **1**, which incorporates the modified residue 4-OH-Pro, is rather unusual.

Iturin F_2_ (**2**) was obtained as a white amorphous powder, whose molecular formula, which is the same as **1**, was determined as C_51_H_80_N_12_O_15_ on the basis of HR-ESIMS in combination with the 1D NMR spectroscopic data (Table 1). Compared to the ^1^H and ^13^C NMR spectra of **1**, noticeable differences were observed in the resonances for two methyls (δ_C/H_ 11.2/0.82 and 19.1/0.82). Further analysis of the 2D NMR spectroscopic data, including the crucial COSY correlations from the methyl H_3_-50 (δ_H_ 0.82) through the aliphatic methylene H_2_-49 (δ_H_ 1.24 and 1.09) and the methine H-48 (δ_H_ 1.27) to the methyl H_3_-51 (δ_H_ 0.82), readily confirmed that **2** contained an *anteiso*-type of β-amino fatty acid chain (Figure 2) [3]. This conclusion was also supported by HMBC correlations of two methyls H_3_-50 and H_3_-51 to the methine C-48 (δ_C_ 33.7) and the methylene C-49 (δ_C_ 28.9). Thus, the structure of compound **2**, which is the positional isomer of **1**, was determined as shown in Figure 1.

Iturin A_9_ (**3**) was isolated as a white amorphous powder. Its molecular formula was assigned as C_51_H_80_N_12_O_14_ on the basis of HR-ESIMS in combination with the 1D NMR spectroscopic data (Table 1). A 16 amu decrease in the molecular weight and a longer reversed-phase HPLC retention time relative to **1** and **2** suggested the loss of the hydroxy group in **3**. The ^13^C NMR spectrum of **3** showed a close similarity to that of **1**, except for the disappearance of an oxymethine signal for C-25. This observation, together with analysis of the 2D NMR spectra, suggested that an oxymethine was replaced by a methylene (δ_C/H_ 24.6/2.00 and 1.88), thus indicating the presence of Pro in **3** rather than 4-OH-Pro. The peptide backbone of **3** was identical to that of iturin A isomers; however, it possessed an *iso*-C_17_ type of β-amino fatty acid chain, which was not previously reported.

**Figure 3:**
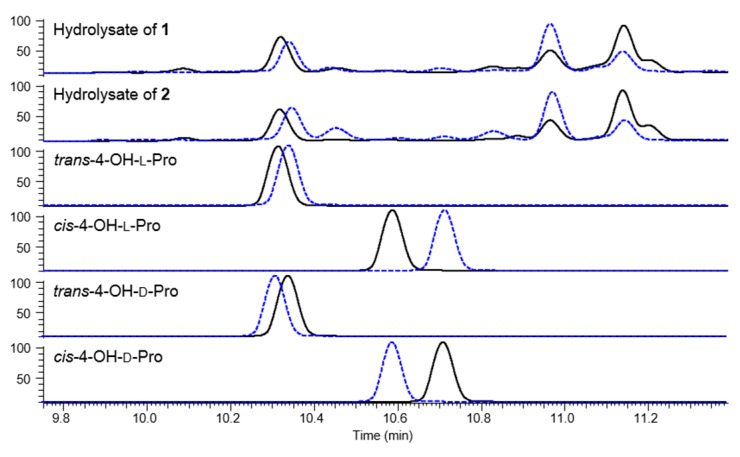
HPLC traces of FDLA derivatives of hydrolysate of **1** and **2** and standard amino acids (black solid line for l-FDLA derivative; blue dashed line for d-FDLA derivatives).

The stereochemistry of **1**–**3** was defined by MS-detected chromatographic comparison of the derivatives of the acid hydrolysate [14]. Advanced Marfey’s method using 1-fluoro-2,4-dinitro-phenyl-5-l/d-leucinamide (l- and d-FDLA) assigned the absolute configurations of the amino acid residues as l-Asn, d-Tyr, d-Asn (×2), l-Gln and l-Ser in **1**–**3** and l-Pro in **3** (Supplementary Information). Moreover, the absolute configurations of α- and γ-carbons of 4-OH-Pro residues in **1** and **2** were elucidated by chromatographic comparisons with FDLA derivatives of four standard residues (Figure 3). The FDLA derivatives of the 4-OH-Pro residue in **1** and **2** showed identical retention times to those of *trans*-4-OH-l-Pro, thus confirming 23*S* and 25*R* configurations.

**Figure 4:**
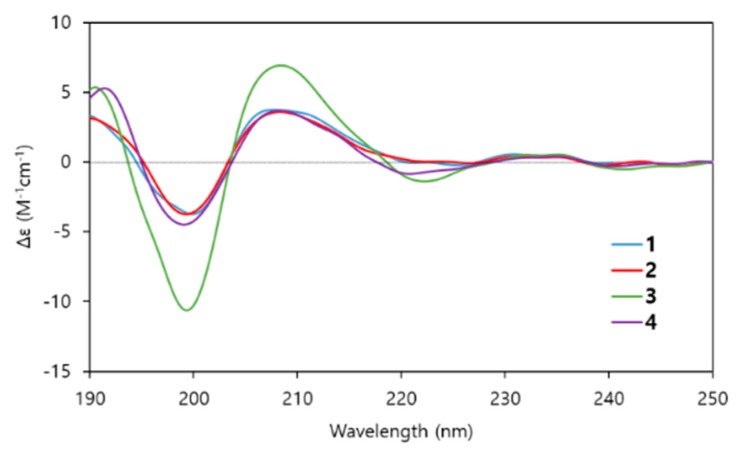
CD spectra of compounds **1**–**4**.

The absolute configuration at C-35 of the β-amino fatty acid chains in **1**–**3** was determined to be *S* on the basis of the elution order of the β-amino fatty acid chain derivatized with FDLA (Supplementary Information) [15,16]. The resulting configuration as 35*R* was in agreement with that of the known iturin class previously assigned by CD spectroscopy [17]. Next, differentiation of l-Asn and d-Asn in the sequence was suggested by means of a conformational analysis based on CD effects. The conformation of iturin A has been investigated by various techniques using NMR spectroscopy, energy calculations, IR and CD spectra [18,19]. Conformational studies based on CD spectra revealed that iturin A in trifluoroethanol showed two positive Cotton effects at 190 and 210 nm, and a negative Cotton effect at 198 nm. To confirm the stereochemical similarity between iturin A and compounds **1**–**3**, CD spectra of these compounds in trifluoroethanol were measured and compared with that of iturin A in the literature. The CD spectra of **1**–**3** showed positive Cotton effects at 190 and 208 nm and a negative Cotton effect at 198 nm, and these are in very good agreement with those of iturin A (Figure 4). Thus, the absolute configurations of Asn_1_, Asn_2_ and Asn_3_ were determined to be l-, d- and d-Asn, respectively, which are the same as those of iturin A. In addition, the structure of compound **4** was established by spectroscopic data, Marfey’s analysis and CD spectra, and consequently determined to be iturin A_8_ bearing an *anteiso*-C_17_-type β-amino fatty acid chain [20].

### 2.3. The Bioactivities of Compounds **1**–**4**

**Figure 5:**
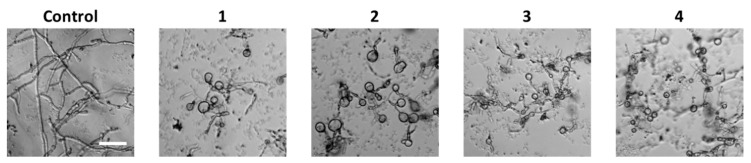
Compounds **1**–**4** showed swelling morphology against *P. oryzae*. *P*. *oryzae* was treated with compounds **1**–**4** at the concentration of 3 µM. Representative images of morphological changes observed under a microscope at 48 h after treatment. Scale bars, 50 μm.

Previous studies have shown that the bioactivities of iturins depend on the composition of the peptide residues and on the type and length of the fatty acid chain [21]. In order to investigate the effects of a hydroxy group at C-25 and the type of fatty acid chain on biological properties of the iturin class, compounds **1**–**4** were subjected to several preliminary tests including antifungal, antibacterial and cytotoxic assays as well as an enzyme-based assay. All tested compounds exhibited antifungal activities against various pathogenic fungi (Table 2). In particular, compounds **1** and **2**, bearing a *trans*-4-OH-l-Pro residue, showed better activities than **3** and **4** against *A*. *flavus*, *N*. *crassa*, *C*. *albicans* and *P*. *griseofulvum*. The fungitoxicity of iturins is known to rely on membrane permeabilization properties by the formation of ion-conducting pores [22]. Microscopic analysis revealed that compounds **1**–**4** showed swelling morphology against *P*. *oryzae* (Figure 5), which might be caused by osmotic perturbations through the interaction of the compounds with the cytoplasmic membrane [2].

A recent study revealed that iturin A inhibits the proliferation of MDA-MB-231 and MCF-7 breast cancer cells by inhibiting the Akt signaling network, suggesting the potential anti-cancer effect of iturin A [23]. In our preliminary cytotoxic evaluation of compounds **1**–**4** against human cervical cancer cells (HeLa) and rat kidney cells infected with a temperature-sensitive mutant of Rous sarcoma virus (*src*^ts^-NRK), all compounds exhibited moderate cytotoxicities toward both cell lines (Table 3), implying a broad spectrum of the cytotoxicity of iturins.

In the antibacterial bioassays using *Staphylococcus aureus* and *Escherichia coli*, they were inactive up to 30 μM, which is consistent with previous bioassay results of known iturins [1]. In order to investigate new biological properties of iturins, compounds **1**–**4** were tested for their ability to inhibit indoleamine 2,3-dioxygenase (IDO). IDO, a promising target for anti-cancer therapy, is expressed by tumor cells and catalyzes the initial and rate-limiting step of the kynurenine pathway, which leads to the production of kynurenine metabolites and tryptophan depletion. These consequently lead to local immunosuppression which allows tumor cells to escape an immune response [24,25,26]. Thus, IDO inhibitors are considered potential anti-cancer agents. Interestingly, despite significant structural similarities, compound **4** showed inhibitory activity in an *in vitro* IDO assay with an IC_50_ value of 5.5 μM (positive control menadione = 0.64 μM), while **1**–**3** were inactive up to 50 μM. To the best of our knowledge, this is the first report of a polypeptide molecule exhibiting IDO-inhibitory activity.

## 3. Materials and Methods

### 3.1. General Experimental Procedures

Optical rotations were measured using a JASCO P-1020 polarimeter, and UV spectra were obtained on an Optizen 2120 UV spectrophotometer. CD spectra were measured on a JASCO J-715 spectropolarimeter. NMR spectra were recorded on a Bruker Biospin Advance II 900 NMR spectrometer (900 MHz for ^1^H and 225 MHz for ^13^C), Bruker AVANCE HD 800 NMR spectrometer (800 MHz for ^1^H and 200 MHz for ^13^C) and Bruker AVANCE HD 700 NMR spectrometer (700 MHz for ^1^H and 175 MHz for ^13^C) at Korea Basic Science Institute (KBSI) in Ochang, Korea. NMR spectra were recorded in DMSO-*d*_6_ and chemical shifts were referenced to residual solvent signal (δ_C_ 39.5, δ_H_ 2.50). High resolution electrospray ionization mass spectrometry (HR-ESIMS) data were acquired on a Q-TOF mass spectrometer (SYNAPT G2, Waters) at KBSI in Ochang, Korea. ODS (75 μm; Cosmosil, Nacalai Tesque, Kyoto, Japan) was used for vacuum liquid chromatography with LP-grade solvents (SK chemicals, Osan, Korea). Analytical C_18_ (4.6 × 150 mm, 5 μm; YMC, Kyoto, Japan), semi-preparative C_18_ (10 × 250 mm, 10 μm; Optimapak, RStech, Daejeon, Korea) columns were used for reversed-phase HPLC on a YL9100 HPLC system (Young Lin, Anyang, Korea) equipped with a YL9160 PDA detector (Young Lin) using HPLC-grade solvents (Burdick & Jackson, Muskegon, MI, USA). A liquid chromatography-mass spectrometry (LC-MS) was performed using an LTQ XL linear ion trap (Thermo Scientific, Rockford, IL, USA) equipped with an electrospray ionization (ESI) source that was coupled to a rapid separation LC (RSLC; ultimate 3000, Thermo Scientific) system (ESI-LC-MS) using a HSS T3 column (2.1 × 150 mm, 2.5 μm; Waters, Milford, MA, USA) with a linear gradient of the binary solvent system consisting of water with 0.1% formic acid and acetonitrile at a flow rate of 0.3 mL/min. A linear gradient was initiated with 5% acetonitrile and linearly increased to 100% at 0–15 min. The ESI parameters were the source voltage (+5 kV), entrance capillary voltage (+18 V), entrance capillary temperature (275 °C) and tube lens voltage (+120 V). The scan range was fixed from *m/z* 50 to 2000. The data-dependent mass spectrometry experiments were controlled using the menu-driven software provided with the Xcalibur system (Thermo Scientific).

### 3.2. Isolation of the Strain KCB14S006

The strain KCB14S006 was isolated from a saline water sample, which was collected from a saltern in Incheon, Korea. Samples were directly diluted to 10^−3^ with artificial sea water and were spread on various solid media plates using a disposable plastic rod. The plates were incubated at 28 °C for four weeks. Strain KCB14S006 was observed from a glycerol-asparagine agar plate (1 g of asparagine, 10 g of glycerol, 1 g of K_2_HPO_4_, 0.5 g of MgSO_4_, 18 g of agar per 1 L of artificial sea water) supplemented with cycloheximide at 100 μg/mL, and purified by transfer on marine agar (Difco Laboratories, Detroit, MI, USA). On the basis of phylogenetic characterization by 16S rRNA gene sequence similarity, strain KCB14S006 was shown to be closely related to *Bacillus licheniformis* ATCC 14580 (99.3%). The strain was deposited at the Anticancer Agent Research Center, KRIBB, Korea, under the curatorship of J.-H.J.

### 3.3. Culture, Extraction and Isolation

For the preparative-scale culture, *Bacillus* sp. KCB14S006 was cultured in 32 square petri dishes by direct streaking, with each containing 75 mL of marine agar (Difco Laboratories). These were continued to be cultured for one week until the bacterium covered the entire surface of the medium. The agar medium containing bacterial colonies was cut into squares and soaked in acetone overnight. After evaporation of the acetone *in vacuo*, the resulting material was successively suspended in H_2_O and partitioned three times with EtOAc. The organic layer was evaporated *in vacuo* to yield a yellow powder (350 mg), which was subjected to reversed-phase C_18_ flash column chromatography with a stepwise solvent system of MeOH:H_2_O (20:80, 40:60, 60:40, 80:20 and 100:0 MeOH, v/v, each 2 × 500 mL). The first 100% aqueous MeOH fraction (29.5 mg) was further purified by reversed-phase HPLC (flow rate: 3 mL/min) using a linear gradient elution of CH_3_CN:H_2_O (35:65–52:48) over 35 min to yield the pure compounds, **1** (*t*_R_: 29.2 min, 2.9 mg), **2** (*t*_R_: 28.4 min, 3.4 mg), **3** (*t*_R_: 31.1 min, 2.3 mg) and **4** (*t*_R_: 30.4 min, 2.4 mg).

Iturin F_1_ (**1**): white amorphous powder; ${\left[\alpha \right]}_{D}^{19}$ +9.5 (*c* 0.05, MeOH); UV (MeOH) λ_max_ (log ε) 204 (4.3), 226 (3.9) nm; for ^1^H and ^13^C NMR spectroscopic data, see Table 1; HR-ESIMS *m/z* 1123.5757 [M + Na]^+^ (calcd for C_51_H_80_N_12_O_15_Na, 1123.5764).

Iturin F_2_ (**2**): white amorphous powder; ${\left[\alpha \right]}_{D}^{18}$ +13.4 (*c* 0.05, MeOH); UV (MeOH) λ_max_ (log ε) 204 (4.3), 226 (3.9) nm; for ^1^H and ^13^C NMR spectroscopic data, see Table 1; HR-ESIMS *m/z* 1123.5758 [M + Na]^+^ (calcd for C_51_H_80_N_12_O_15_Na, 1123.5764).

Iturin A_9_ (**3**): white amorphous powder; ${\left[\alpha \right]}_{D}^{19}$ +13.6 (c 0.05, MeOH); UV (MeOH) λ_max_ (log ε) 205 (4.3), 227 (3.9) nm; for ^1^H and ^13^C NMR spectroscopic data, see Table 1; HR-ESIMS *m/z* 1107.5808 [M + Na]^+^ (calcd for C_51_H_80_N_12_O_14_Na, 1107.5815).

Iturin A_8_ (**4**): white amorphous powder; ${\left[\alpha \right]}_{D}^{19}$ +3.5 (*c* 0.05, MeOH); UV (MeOH) *λ*_max_ (log ε) 204 (4.3), 226 (3.9) nm; HR-ESIMS *m/z* 1107.5807 [M + Na]^+^ (calcd for C_51_H_80_N_12_O_14_Na, 1107.5815).

### 3.4. Antibacterial Assay

The following microorganisms were used as test strains in the assay; *Staphylococcus aureus* 209 and *Escherichia coli* HO141. Antimicrobial activities of compounds against these microorganisms were conducted by a standard microdilution method. Briefly, 100 μL of cell suspension containing 0.1% of a 0.5 McFarland standard suspension was plated into 96-well plate. Test compounds were added to the culture medium, and the plates were incubated at 28 °C (*S*. *aureus*) or 37 °C (*E*. *coli*) for 24 h. The growths of these microorganisms were measured by absorbance at 600 nm.

### 3.5. Antifungal Assay

The following microorganisms were used as test strains in the assay; *Aspergillus* *flavus* KCTC 6984, *Neurospora crassa* KCTC 6079, *Candida tropicalis* KCTC 7830, *Candida albicans* KCTC 7678, *Fusarium oxysporum* f. sp. cucumerinum KCTC 6084, *Alternaria brassicicola* ATCC 96836 and *Penicillium griseofulvum* KCTC 6435. Potato dextrose broth (Difco Laboratories) was used for a test medium. For *C*. *albicans* and *C*. *tropicalis*, 100 μL of inoculum suspension of 10^5^ spores/mL was plated into 96-well plates containing diluted test compounds, and the plates were incubated at 28 °C for 48 h. For *A*. *flavus*, *N*. *crassa*, *F*. *oxysporum*, *A*. *brassicicola* and *P*. *griseofulvum*, 100 μL of cell suspension containing 2% of overnight cultures in 96-well were incubated at 28 °C for 72 h. The MIC values were determined as the lowest concentration of each compound that prevented visible growth.

### 3.6. Cytotoxicity Assay

The human cervix epidermoid carcinoma cell line HeLa was cultured at 37 °C in DMEM (Invitrogen/Life Technologies, Carlsbad, CA, USA), supplemented with 10% fetal bovine serum (Sigma-Aldrich, St. Louis, MO, USA). *src*^ts^-NRK cells, rat kidney cells that were infected with ts25, a T-class mutant of Rous sarcoma virus Prague strain, were cultured at permissive temperature (32 °C) in MEM (Sigma-Aldrich), supplemented with 10% calf serum (PAA Laboratories, Pashing, Austria). Each cell line was seeded into a 96-well plate (HeLa, 4 × 10^3^ cells/well; *src*^ts^-NRK, 1 × 10^4^ cells/well) and then exposed to test compounds. After 48 h incubation, cell proliferation was determined using a Cell Count Reagent SF (Nacalai Tesque). Briefly, a 1/10 volume of WST-8 solution was added to each well, and the plates were incubated at 37 °C for 1 h. Then, the absorbance at 450 nm was measured in a microplate reader.

### 3.7. Indoleamine 2,3-Dioxygenase (IDO) Inhibition Assay

The enzymatic inhibition assay was performed with human recombinant IDO expressed in *E*. *coli* according to previously described method with some modifications [27]. The reaction mixture (200 μL) contained 50 mM potassium phosphate buffer (pH 6.5), 20 mM ascorbic acid, 10 μM methylene blue, 200 μM L-Trp, 20 μg/mL catalase and a sample in 2 μL of DMSO. The samples were serially diluted 10-fold from 50 to 0.1 μM. The reaction was started by the addition of IDO and continued at 37 °C for 60 min. After termination of the reaction by addition of 40 μL of 30% (w/v) trichloroacetic acid, the resulting mixtures were incubated at 60 °C for 15 min, followed by centrifugation at 2000 × *g* for 15 min. Finally, 125 μL of supernatant is added to 125 μL of *p*-dimethylaminobenzaldehyde (*p*DMAB) (2%, v/v) in acetic acid, and the absorbance at 480 nm was measured.

### 3.8. Advanced Marfey’s Analysis

Compounds **1**–**4** (each 0.1 mg) were each dissolved in 0.5 mL of 6 N HCl and shaken at 100 °C for 1 h. After excess HCl was removed under vacuum, the reaction product was dissolved in 100 μL of H_2_O and separated into two portions. The residue was then dried and dissolved in 100 μL of 1 N NaHCO_3_. Either 100 μL of 1% l- or d-FDLA in acetone was added to each hydrolysate, and the resulting mixture was vortexed and heated at 40 °C for 1 h. The reaction was quenched by the addition of 20 μL of 2 N HCl and 20 μL of the reaction mixture was diluted with 20 μL of CH_3_CN. The solution was analyzed by LC-MS. Standard amino acids were also derivatized with FDLA in the same manner and were subjected to LC-MS to compare retention times of reaction products.

## 4. Conclusions

Chemical investigation of *Bacillus* sp. KCB14S006 obtained from salterns has led to the isolation of three new iturin class lipopeptides (**1**–**3**), along with one known metabolite, iturin A_8_ (**4**). The absolute configurations including a newly identified 4-OH-Pro residue were determined by Marfey’s analysis and CD spectroscopy. All isolates showed antifungal activities with slight differences due to the presence or absence of the hydroxy group in Pro and cytotoxic activities. In particular, compound **4** was found to show IDO-inhibitory activity with an IC_50_ value of 5.5 μM. Structurally diverse secondary metabolites, including lipopeptides, polypeptides, macrolactones and polyketides, have been continuously discovered from marine-derived *Bacillus* strains [28,29,30,31]. However, relatively few metabolites have been characterized from *Bacillus* sp. inhabiting hypersaline environments, such as saline lakes and salterns where the salt concentration is much higher than that of ocean [32,33]. The discovery of the iturins and their bioactivities presented here supports the use of bacteria from salterns as promising sources of bioactive small molecules, and helps to understand the diversity of the iturin class which possesses various bioactivities.

## Figures and Tables

**Table 1:** NMR spectroscopic data for compounds **1**–**3** in DMSO-*d*_6_.

Position	1 ^1^		2 ^2^		3 ^3^	
	δ_C_	δ_H_, m (*J* in Hz)	δ_C_	δ_H_, m (*J* in Hz)	δ_C_	δ_H_, m (*J* in Hz)
1	50.9	4.39, ovl	50.9	4.39, ovl	50.8	4.43, ovl
2	36.2	2.30, dd (15.9, 8.8)	36.2	2.29, m	36.3	2.29, dd (15.7, 8.4)
		2.13, dd (15.9, 4.6)		2.13, dd (16.3, 5.0)		2.16, dd (15.7, 5.4)
3	170.5		170.6		170.6	
4	173.4		173.4		173.4	
1-NH		7.78, s		7.78, d (4.2)		7.72, d (4.1)
3-NH_2_		7.34, s		7.35, s		7.33, ovl
		6.92, ovl		6.91, ovl		6.92, s
5	56.3	4.03, m	56.3	4.02, m	56.3	4.02, m
6	34.9	2.97, br d (10.6)	34.9	2.97, dd (14.5, 3.5)	34.9	2.96, dd (14.3, 3.4)
		2.75, dd (14.1, 10.6)		2.74, dd (14.5, 11.4)		2.75, dd (14.3, 10.5)
7	128.0		128.0		128.0	
8	129.8	7.03, d (8.4)	129.8	7.03, d (8.8)	129.8	7.03, d (8.4)
9	115.1	6.66, d (8.3)	115.1	6.66, d (8.7)	115.1	6.66, d (8.4)
10	155.8		155.8		155.8	
11	115.1	6.66, d (8.3)	115.1	6.66, d (8.7)	115.1	6.66, d (8.4)
12	129.8	7.03, d (8.4)	129.8	7.03, d (8.8)	129.8	7.03, d (8.4)
13	171.3		171.3		171.2	
5-NH		8.69, s		8.69, d (6.3)		8.72, ovl
10-OH		9.21, s		9.21, s		9.19, s
14	50.9	4.48, m	50.9	4.48, m	49.6	4.43, ovl
15	36.2	2.59, dd (15.5, 10.0)	36.2	2.59, dd (15.6, 10.6)	36.0	2.59, dd (15.5, 9.6)
		2.50, ovl		2.50, ovl		2.49, ovl
16	171.1		171.1		171.2	
17	171.2		171.2		171.1	
14-NH		8.07, d (7.1)		8.07, d (8.2)		8.06, d (6.6)
16-NH_2_		7.21, ovl		7.21, s		7.22, s
		6.89, ovl		6.89, ovl		6.88, s
18	49.6	4.51, dd (14.1, 8.6)	49.6	4.51, m	49.6	4.51, dd (14.1, 8.5)
19	26.7	2.00, m	26.7	1.99, ovl	26.5	2.02, ovl
		1.71, m		1.72, m		1.74, ovl
20	30.5	2.09, ovl	30.5	2.09, ovl	30.6	2.10, ovl
21	174.0		174.0		174.1	
22	171.2		171.1		170.9	
18-NH		6.92, ovl		6.92, ovl		6.98, d (7.8)
21-NH_2_		7.10, s		7.11, s		7.13, s
		6.85, ovl		6.85, ovl		6.86, s
23	59.7	4.28, t (7.9)	59.7	4.28, t (8.7)	60.1	4.17, ovl
24	37.5	2.03, m	37.5	2.03, m	29.0	2.13, ovl
		1.86, m		1.86, m		1.77, m
25	68.7	4.39, ovl	68.7	4.39, ovl	24.6	2.00, ovl
						1.88, m
26	55.7	3.82, br d (6.9)	55.6	3.82, dd (10.1, 3.4)	47.2	3.78, m
		3.69, ovl		3.69, ovl		3.74, m
27	172.6		172.6		172.7	
25-OH		5.22, s		5.23, d (2.9)		
28	49.7	4.43, m	49.7	4.43, m	49.7	4.43, ovl
29	35.2	2.70, dd (15.5, 5.0)	35.2	2.70, dd (16.5, 5.7)	35.2	2.71, dd (15.7, 5.4)
		2.43, dd (15.5, 7.5)		2.43, dd (16.5, 8.1)		2.47, ovl
30	171.8		171.8		171.8	
31	170.8		170.8		170.8	
28-NH		8.79, d (6.8)		8.80, d (8.0)		8.72, ovl
30-NH_2_		7.37, s		7.38, s		7.38, s
		6.85, ovl		6.85, ovl		6.85, s
32	56.3	4.17, dd (11.7, 7.0)	56.3	4.17, dd (12.3, 7.3)	56.2	4.17, ovl
33	61.4	3.69, ovl	61.4	3.69, ovl	61.4	3.66, t (5.5)
		3.65, m		3.65, ovl		
34	170.5		170.5		170.3	
32-NH		7.40, s		7.40, ovl		7.33, ovl
33-OH		4.87, t (5.5)		4.88, t (6.0)		4.87, t (5.9)
35	45.2	3.99, m	45.2	3.99, m	45.3	3.98, m
36	41.9	2.35, m	41.9	2.35, m	41.8	2.34, ovl
37	171.3		171.3		171.2	
38	34.6	1.43, m	34.6	1.40, m	34.6	1.41, m
		1.40, m				
39	25.3	1.24, ovl	25.3	1.24, ovl	25.4	1.24, ovl
		1.13, ovl		1.11, ovl		1.13, ovl
40–46	28.6–29.3	1.24, ovl	28.6–26.5	1.24, ovl	28.6–29.3	1.24, ovl
47	26.8	1.24, ovl	36.0	1.26, ovl	26.8	1.24, ovl
		1.13, ovl		1.08, m		1.13, ovl
48	38.5	1.24, ovl	33.7	1.27, ovl	38.5	1.24, ovl
		1.13, ovl				1.13, ovl
49	27.4	1.49, m	28.9	1.24, ovl	27.4	1.49, m
				1.09, ovl		
50	22.5	0.84, d (6.6)	11.2	0.82, ovl	22.6	0.84, d (6.6)
51	22.5	0.84, d (6.6)	19.1	0.82, ovl	22.6	0.84, d (6.6)
35-NH		7.21, ovl		7.21, s		7.14, ovl

**^1^** 900 MHz for ^1^H and 225 MHz for ^13^C NMR data; **^2^** 700 MHz for ^1^H and 175 MHz for ^13^C NMR data; **^3^** 800 MHz for ^1^H and 200 MHz for ^13^C NMR data; **O**vl: overlapped with other signals.

**Table 2:** Antifungal activities of compounds **1**–**4** (MIC, μg/mL).

Fungi	1	2	3	4	Amphotericin B
*Aspergillus flavus*	3.125	3.125	25	12.5	0.195
*Neurospora crassa*	6.25	6.25	12.5	12.5	0.098
*Candida tropicalis*	6.25	6.25	6.25	6.25	0.781
*Candida albicans*	6.25	6.25	12.5	12.5	0.098
*Fusarium oxysporum*	25	25	25	25	12.5
*Alternaria brassicicola*	25	25	25	25	0.391
*Penicillium griseofulvum*	3.125	3.125	6.25	6.25	3.125

**Table 3:** Cytotoxic activities of compounds **1**–**4** (IC_50_, μM).

Cell Line	1	2	3	4
HeLa	8.9	7.8	4.6	5.6
*src*^ts^-NRK	8.9	7.4	5.2	9.0

## Supplementary Files

Supplementary File 1

## References

[1] Ongena M., Jacques P. (2008). *Bacillus* lipopeptides: Versatile weapons for plant disease biocontrol. Trends. Microbiol..

[2] Aranda F.J., Teruel J.A., Ortiz A. (2005). Further aspects on the hemolytic activity of the antibiotic lipopeptide iturin A. Biochim. Biophys. Acta.

[3] Bland J.M. (1996). The first synthesis of a member of the iturin family, the antifungal cyclic lipopeptide, iturin-A2. J. Org. Chem..

[4] Isogai A., Takayama S., Murakoshi S., Suzuki A. (1982). Structure of β-amino acids in antibiotics iturin A. Tetrahedron Lett..

[5] Monciardini P., Iorio M., Maffioli S., Sosio M., Donadio S. (2014). Discovering new bioactive molecules from microbial sources. Microb. Biotechnol..

[6] Fenical W., Jensen P.R. (2006). Developing a new resource for drug discovery: Marine actinomycete bacteria. Nat. Chem. Biol..

[7] Jose P.A., Jebakumar S.R.D. (2014). Unexplored hypersaline habitats are sources of novel actinomycetes. Front. Microbiol..

[8] Wang Y., Zheng J., Liu P., Wang W., Zhu W. (2011). Three new compounds from *Aspergillus terreus* PT06-2 grown in a high salt medium. Mar. Drugs.

[9] Liu H., Xiao L., Wei J., Schmitz J.C., Liu M., Wang C., Cheng L., Wu N., Chen L., Zhang Y. (2013). Identification of *Streptomyces* sp. nov. WH26 producing cytotoxic compounds isolated from marine solar saltern in China. World J. Microbiol. Biotechnol..

[10] Kim S.-H., Ha T.-K.-Q., Oh W.K., Shin J., Oh D.-C. (2016). Antiviral indolosesquiterpenoid xiamycins C-E from a halophilic Actinomycete. J. Nat. Prod..

[11] Scifinder. http://scifinder.cas.org.

[12] Dictionary of Natural Products. http://dnp.chemnetbase.com.

[13] Besson F., Michel G. (1987). Isolation and characterization of new iturins: Iturin D and iturin E. J. Antibiot..

[14] Harada K., Fujii K., Hayashi K., Suzuki M., Ikai Y., Oka H. (1996). Application of d,l-FDLA derivatization to determination of absolute configuration of constituent amino acids in peptide by advanced Marfey’s method. Tetrahedron Lett..

[15] Etzbach L., Plaza A., Garcia R., Baumann S., Müller R. (2014). Cystomanamides: Structure and biosynthetic pathway of a family of glycosylated lipopeptides from Myxobacteria. Org. Lett..

[16] Gerwick W.H., Jiang Z.D., Agarwal S.K., Farmer B.T. (1992). Total structure of hormothamnin A, a toxic cyclic undecapeptide from the tropical marine cyanobacterium *Hormothamnion enteromorphoides*. Tetrahedron.

[17] Nagai U., Besson F., Peypoux F. (1979). Absolute configuration of an iturinic acid as determined by CD spectrum of its DNP-*p*-methoxyanilide. Tetrahedron Lett..

[18] Marion D., Genest M., Caille A., Peypoux F., Michel G., Ptak M. (1986). Conformational study of bacterial lipopeptides: Refinement of the structure of iturin A in solution by two-dimensional ^1^H-NMR and energy calculations. Biopolymers.

[19] Besson F., Raimbault C., Hourdou M.L., Buchet R. (1996). Solvent-induced conformational modifications of iturin A: An infrared and circular dichroic study of a l,d-lipopeptide of *Bacillus subtilis*. Spectrochim. Acta A Mol. Biomol. Spectrosc..

[20] Hiradate S., Yoshida S., Sugie H., Yada H., Fujii Y. (2002). Mulberry anthracnose antagonists (iturins) produced by *Bacillus amyloliquefaciens* RC-2. Phytochemistry.

[21] Maget-Dana R., Peypoux F. (1994). Iturins, a special class of pore-forming lipopeptides: Biological and physicochemical properties. Toxicology.

[22] Bonmatin J.M., Laprévote O., Peypoux F. (2003). Diversity among microbial cyclic lipopeptides: Iturins and surfactins. Activity-structure relationships to design new bioactive agents. Comb. Chem. High Throughput Screen..

[23] Dey G., Bharti R., Dhanarajan G., Das S., Dey K.K., Kumar B.N.P., Sen R., Mandal M. (2015). Marine lipopeptide Iturin A inhibits Akt mediated GSK3β and FoxO3a signaling and triggers apoptosis in breast cancer. Sci. Rep..

[24] Platten M., von Knebel Doeberitz N., Oezen I., Wick W., Ochs K. (2015). Cancer immunotherapy by targeting IDO1/TDO and their downstream effectors. Front. Immunol..

[25] Uyttenhove C., Pilotte L., Théate I., Stroobant V., Colau D., Parmentier N., Boon T., Van den Eynde B.J. (2003). Evidence for a tumoral immune resistance mechanism based on tryptophan degradation by indoleamine 2,3-dioxygenase. Nat. Med..

[26] Liu X., Newton R.C., Friedman S.M., Scherle P.A. (2009). Indoleamine 2, 3-dioxygenase, an emerging target for anti-cancer therapy. Curr. Cancer Drug Targets.

[27] Cady S.G., Sono M. (1991). 1-Methyl-dl-tryptophan, β-(3-benzofuranyl)-dl-alanine (the oxygen analog of tryptophan), and β-[3-benzo(*b*)thienyl]-dl-alanine (the sulfur analog of tryptophan) are competitive inhibitors for indoleamine 2,3-dioxygenase. Arch. Biochem. Biophys..

[28] Mondol M.A., Shin H.J., Islam M.T. (2013). Diversity of secondary metabolites from marine *Bacillus* species: Chemistry and biological activity. Mar. Drugs.

[29] Mondol M.A., Kim J.H., Lee M.A., Tareq F.S., Lee H.S., Lee Y.J., Shin H.J. (2011). Ieodomycins A–D, antimicrobial fatty acids from a marine *Bacillus* sp.. J. Nat. Prod..

[30] Barsby T., Kelly M.T., Andersen R.J. (2002). Tupuseleiamides and basiliskamides, new acyldipeptides and antifungal polyketides produced in culture by a *Bacillus laterosporus* isolate obtained from a tropical marine habitat. J. Nat. Prod..

[31] Pettit G.R., Knight J.C., Herald D.L., Pettit R.K., Hogan F., Mukku V.J., Hamblin J.S., Dodson M.J., Chapuis J.C. (2009). Antineoplastic agents. 570. Isolation and structure elucidation of bacillistatins 1 and 2 from a marine *Bacillus silvestris*. J. Nat. Prod..

[32] Donio M.B., Ronica S.F., Viji V.T., Velmurugan S., Jenifer J.A., Michaelbabu M., Citarasu T. (2013). Isolation and characterization of halophilic *Bacillus* sp. BS3 able to produce pharmacologically important biosurfactants. Asian Pac. J. Trop. Med..

[33] Carballeira N.M., Miranda C., Lozano C.M., Nechev J.T., Ivanova A., Ilieva M., Tzvetkova I., Stefanov K. (2001). Characterization of novel methyl-branched chain fatty acids from a halophilic *Bacillus* species. J. Nat. Prod..

