# Supplementary material for: New Cyclic Lipopeptides of the Iturin Class Produced by Saltern-Derived Bacillus sp. KCB14S006
Source: Mar Drugs. 2016 Apr 2;14(4):72. doi: 10.3390/md14040072 (PMC4849076; doi:10.3390/md14040072)
Supplement: Supplementary File 1 [file marinedrugs-14-00072-s001.pdf]

Supplementary Materials: New Cyclic Lipopeptides of the Iturin Class Produced by Saltern-Derived *Bacillus* sp. KCB14S006

Sangkeun Son, Sung-Kyun Ko, Mina Jang, Jong Won Kim, Gil Soo Kim, Jae Kyoung Lee, Eun Soo Jeon, Yushi Futamura, In-Ja Ryoo, Jung-Sook Lee, Hyuncheol Oh, Young-Soo Hong, Bo Yeon Kim, Shunji Takahashi, Hiroyuki Osada, Jae-Hyuk Jang and Jong Seog Ahn

Table of Contents

**1**	**NMR and HRESIMS Spectra**
Figure S1	^{1}H NMR (900 MHz, DMSO-d_6) spectrum of **1**
Figure S2	^{13}C NMR (225 MHz, DMSO-d_6) spectrum of **1**
Figure S3	HSQC-DEPT spectrum of **1**
Figure S4	DQF-COSY spectrum of **1**
Figure S5	TOCSY spectrum of **1**
Figure S6	HMBC spectrum of **1**
Figure S7	ROESY spectrum of **1**
Figure S8	^{1}H NMR (700 MHz, DMSO-d_6) spectrum of **2**
Figure S9	^{13}C NMR (175 MHz, DMSO-d_6) spectrum of **2**
Figure S10	HSQC-DEPT spectrum of **2**
Figure S11	COSY spectrum of **2**
Figure S12	HMCB spectrum of **2**
Figure S13	ROESY spectrum of **2**
Figure S14	^{1}H NMR (800 MHz, DMSO-d_6) spectrum of **3**
Figure S15	^{13}C NMR (200 MHz, DMSO-d_6) spectrum of **3**
Figure S16	HSQC-DEPT spectrum of **3**
Figure S17	COSY spectrum of **3**
Figure S18	HMBC spectrum of **3**
Figure S19	ROESY spectrum of **3**
Figure S20	HRESIMS spectrum of **1**
Figure S21	HRESIMS spectrum of **2**
Figure S22	HRESIMS spectrum of **3**
**2**	**Chromatographic Comparisons of FDLA Derivatives**
Table S1	Retention times (t_R, min) of FDLA derivatives for **1**–**4**
Table S2	Retention times (t_R, min) of FDLA derivatives for 4-OH-Pro in **1** and **2**, and standard amino acids
Table S3	Retention times (t_R, min) of FDLA derivatives for β-amino fatty acids in **1**–**4**
Figure S23	HPLC traces corresponding to Marfey's analysis of **1**
Figure S24	HPLC traces corresponding to Marfey's analysis of **2**
Figure S25	HPLC traces corresponding to Marfey's analysis of **3**
Figure S26	HPLC traces corresponding to Marfey's analysis of **4**
Figure S27	HPLC traces of L-FDLA derivatives of 4-OH-Pro in **1** and **2**, and standard amino acids
Figure S28	HPLC traces of D-FDLA derivatives of 4-OH-Pro in **1** and **2**, and standard amino acids
Figure S29	HPLC traces of L- and D-FDLA derivatives of fatty acid chains in **1**–**4**

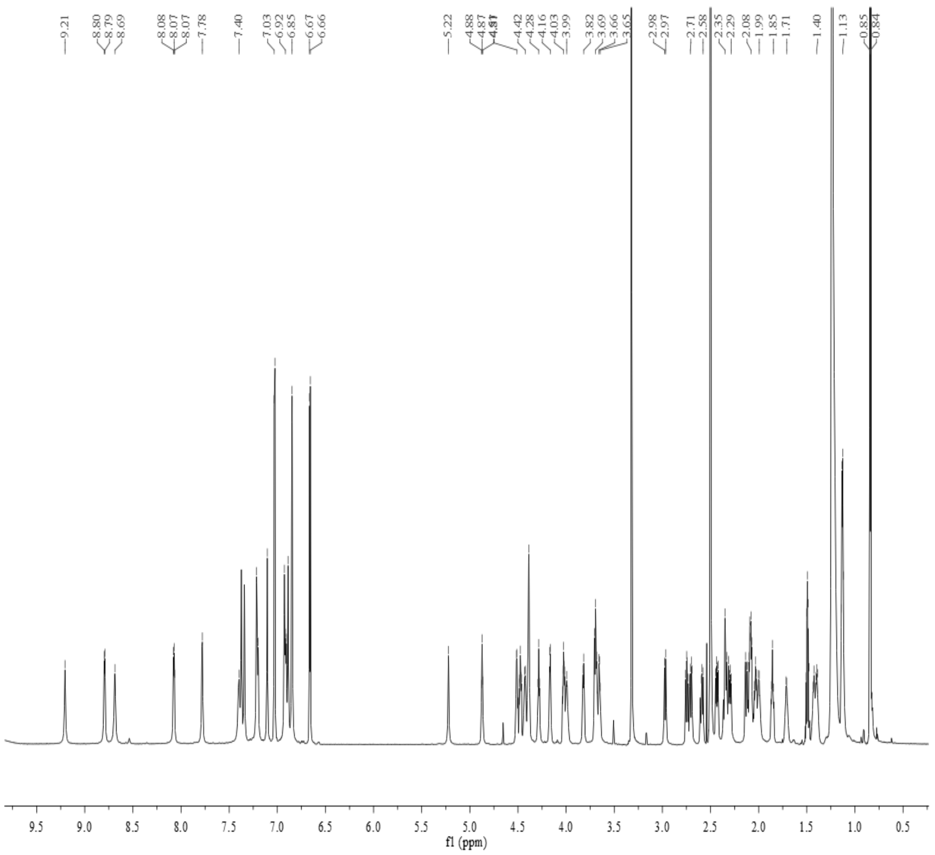

Figure S1. ^{1}H NMR (900 MHz, DMSO-*d*6) spectrum of **1**.

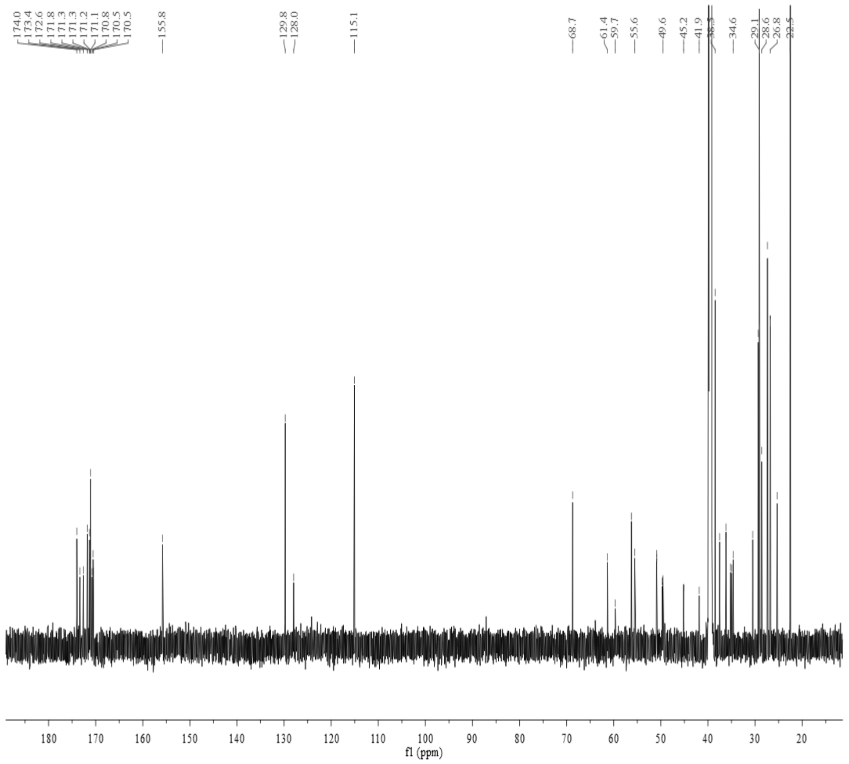

Figure S2. ^{13}C NMR (225 MHz, DMSO-*d*6) spectrum of **1**.

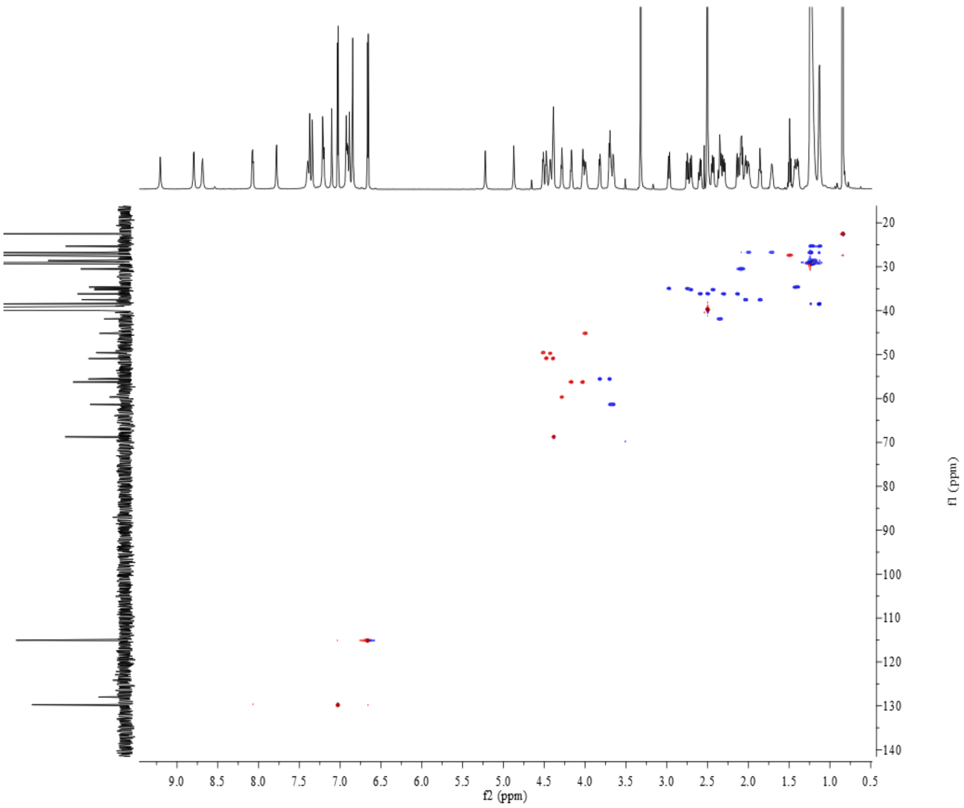

Figure S3. HSQC-DEPT spectrum of **1**

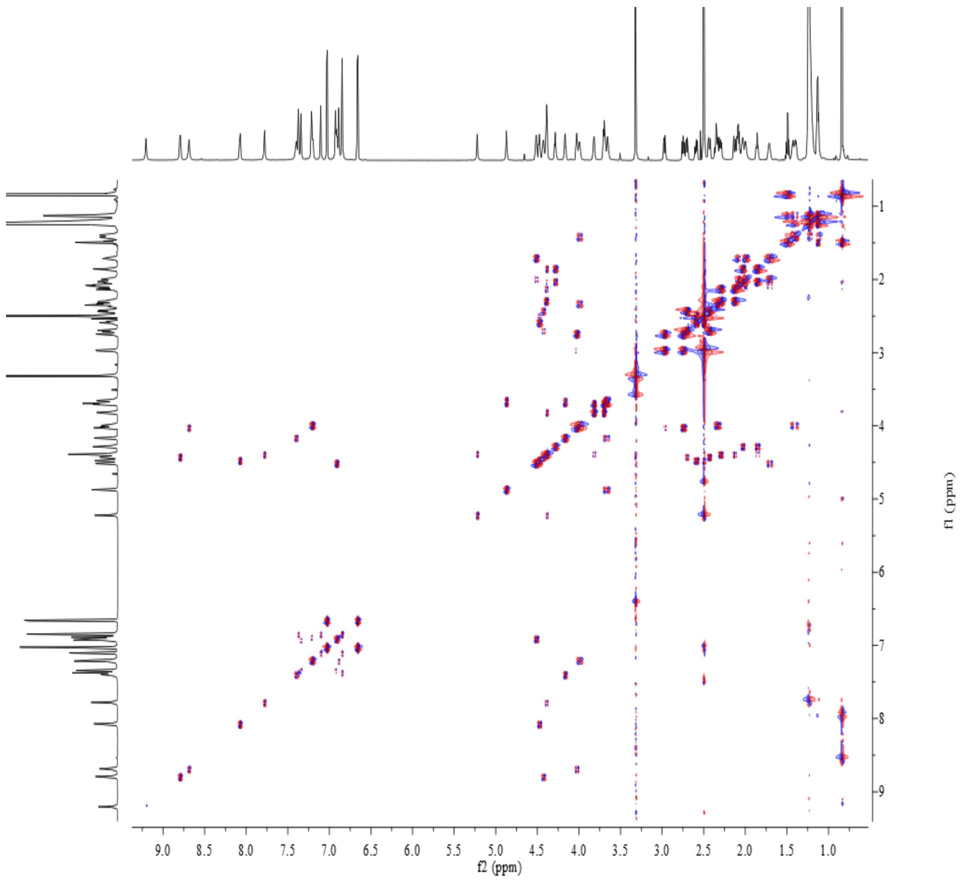

Figure S4. DQF-COSY spectrum of **1**.

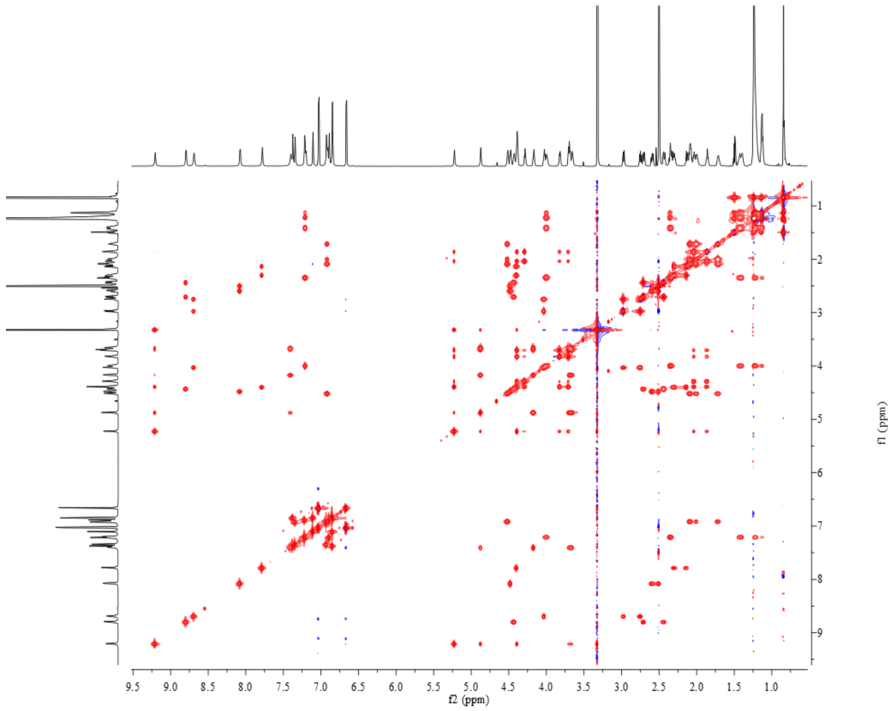

Figure S5. TOCSY spectrum of **1**.

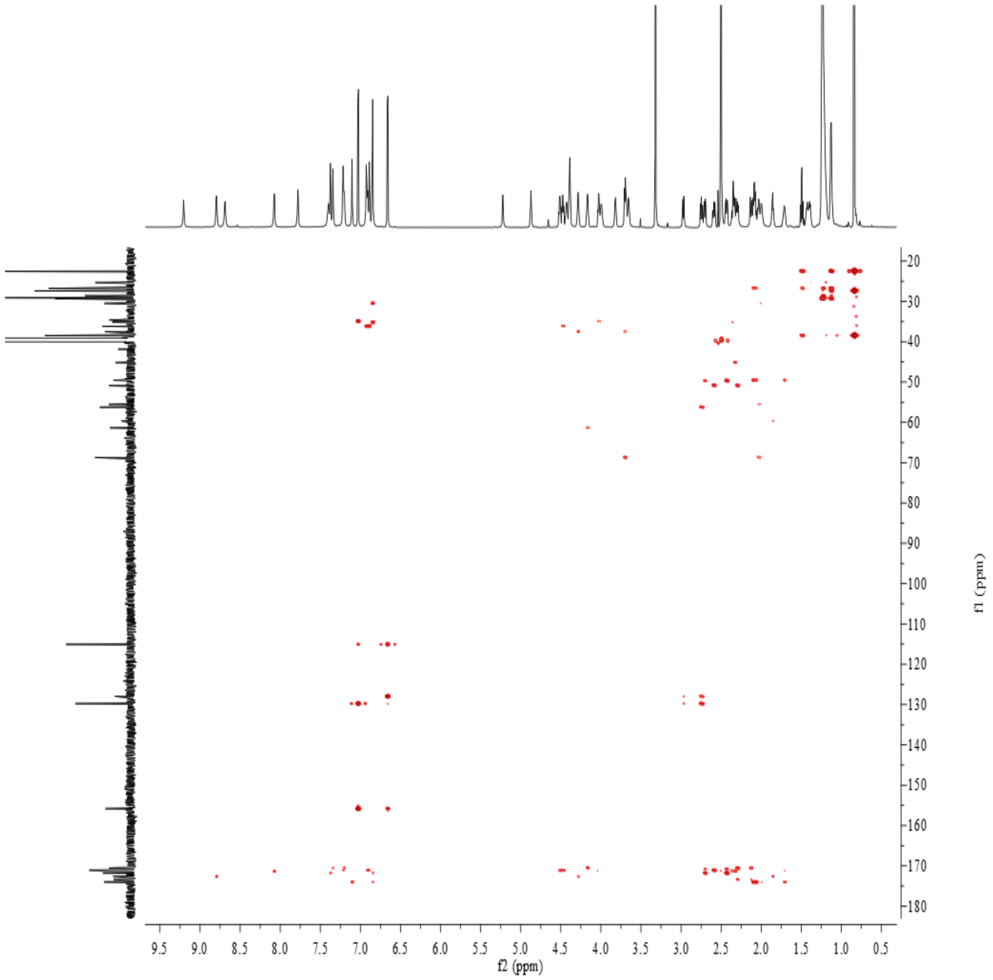

Figure S6. HMBC spectrum of **1**.

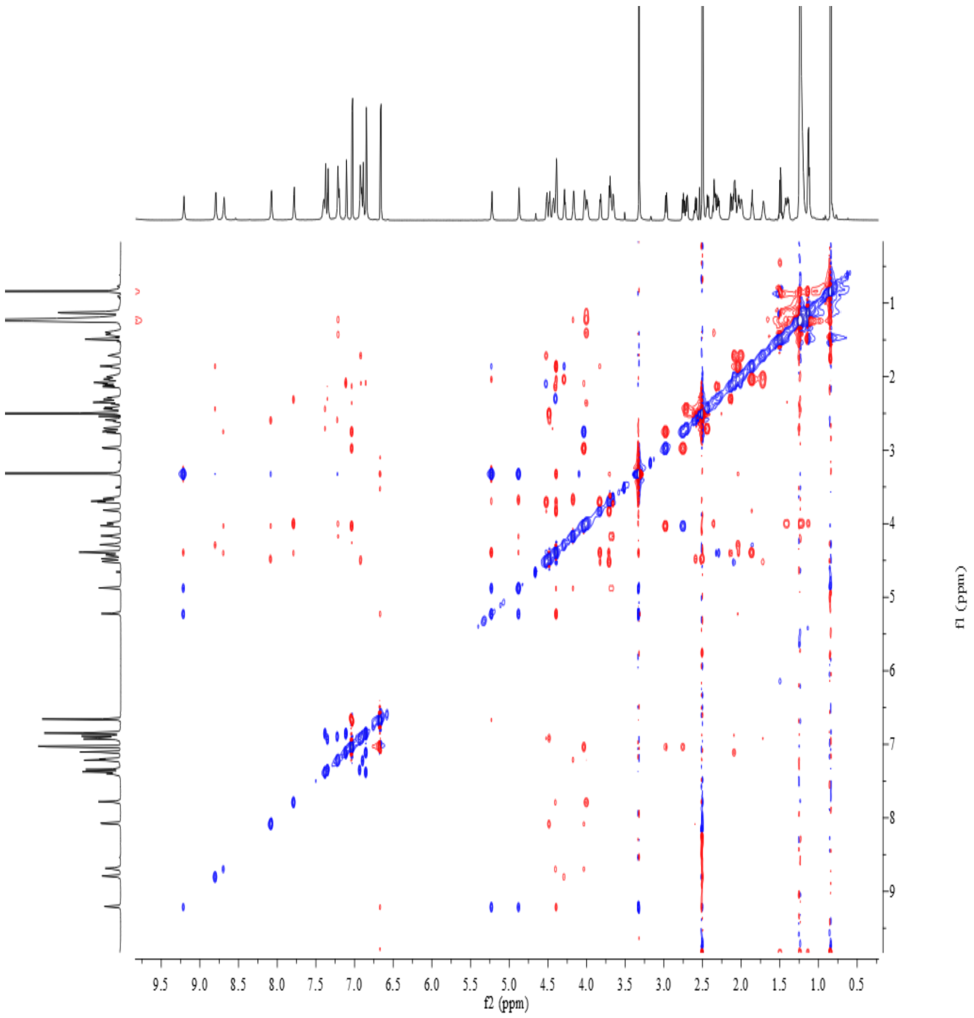

Figure S7. ROESY spectrum of **1**.

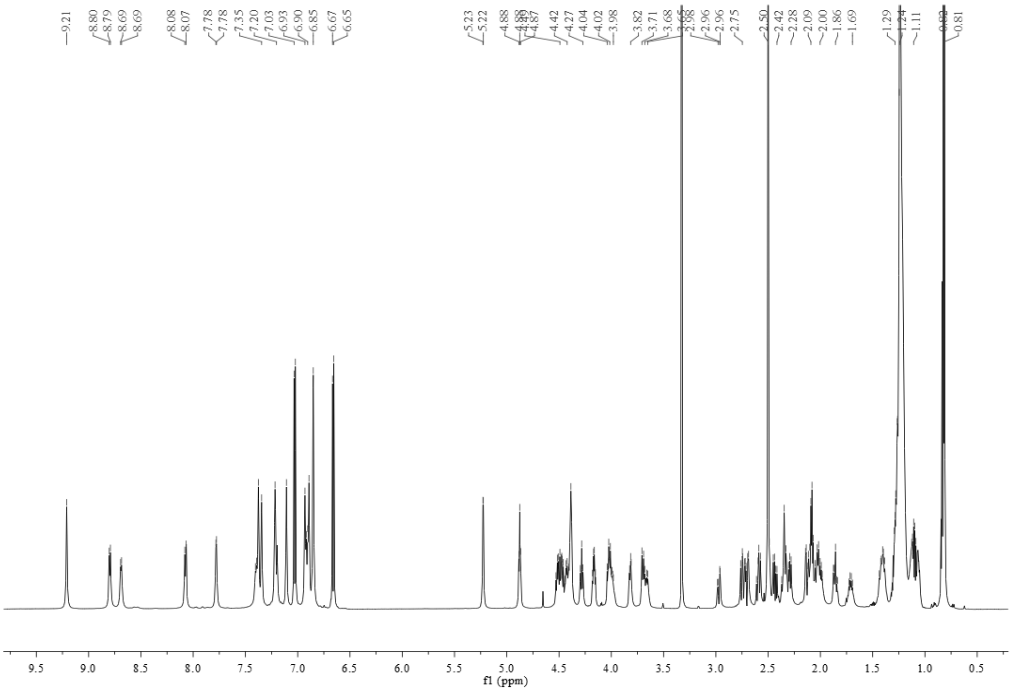

Figure S8. ^{1}H NMR (700 MHz, DMSO-*d*6) spectrum of **2**.

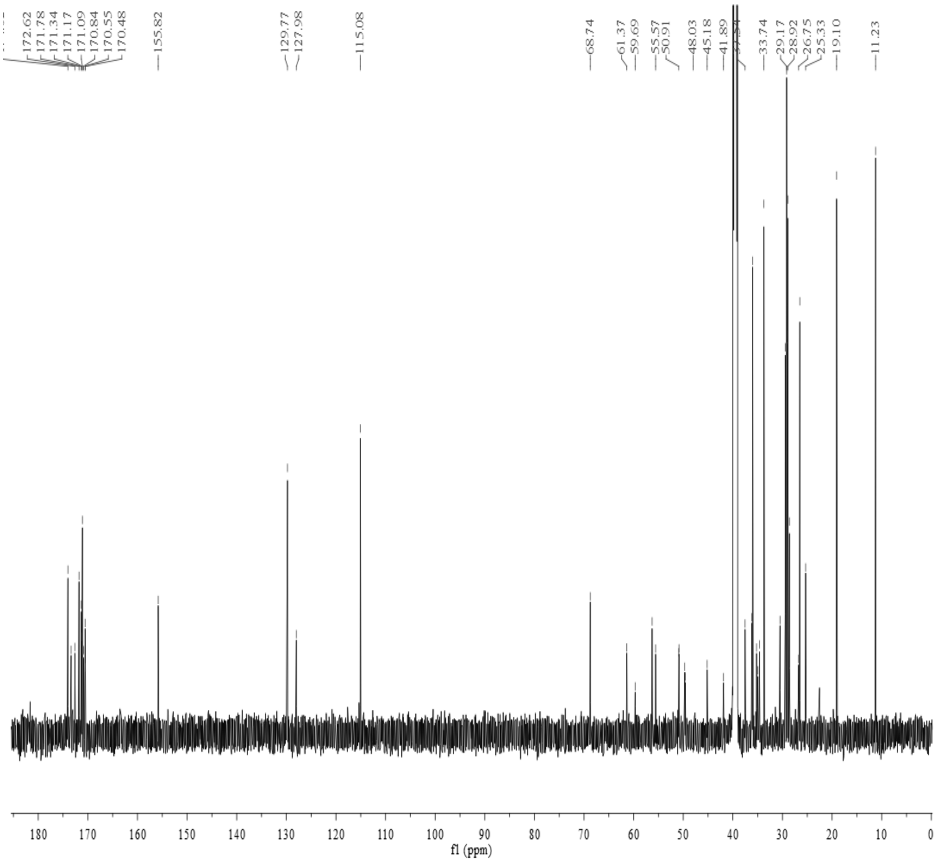

Figure S9. ^{13}C NMR (175 MHz, DMSO-*d*6) spectrum of **2**.

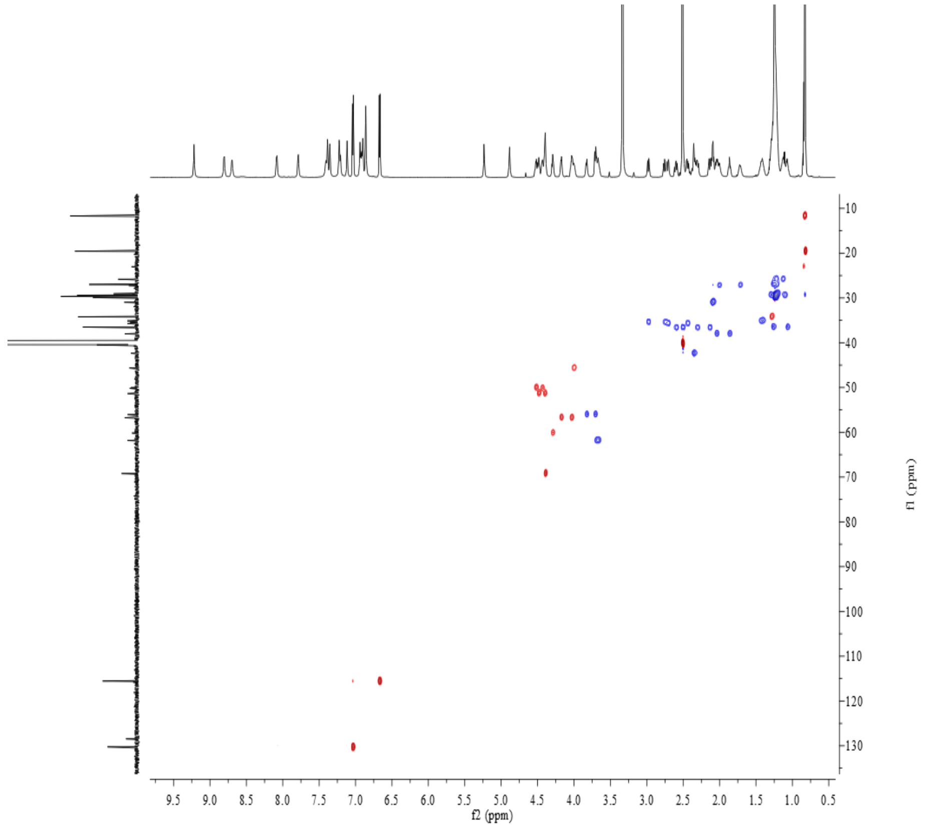

Figure S10. HSQC-DEPT spectrum of **2**.

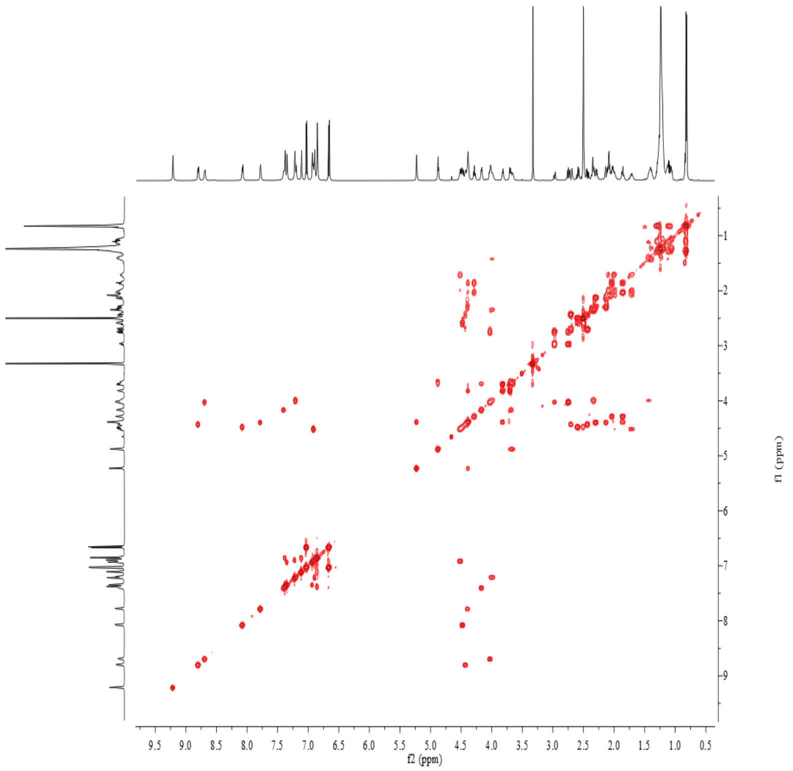

Figure S11. COSY spectrum of **2**.

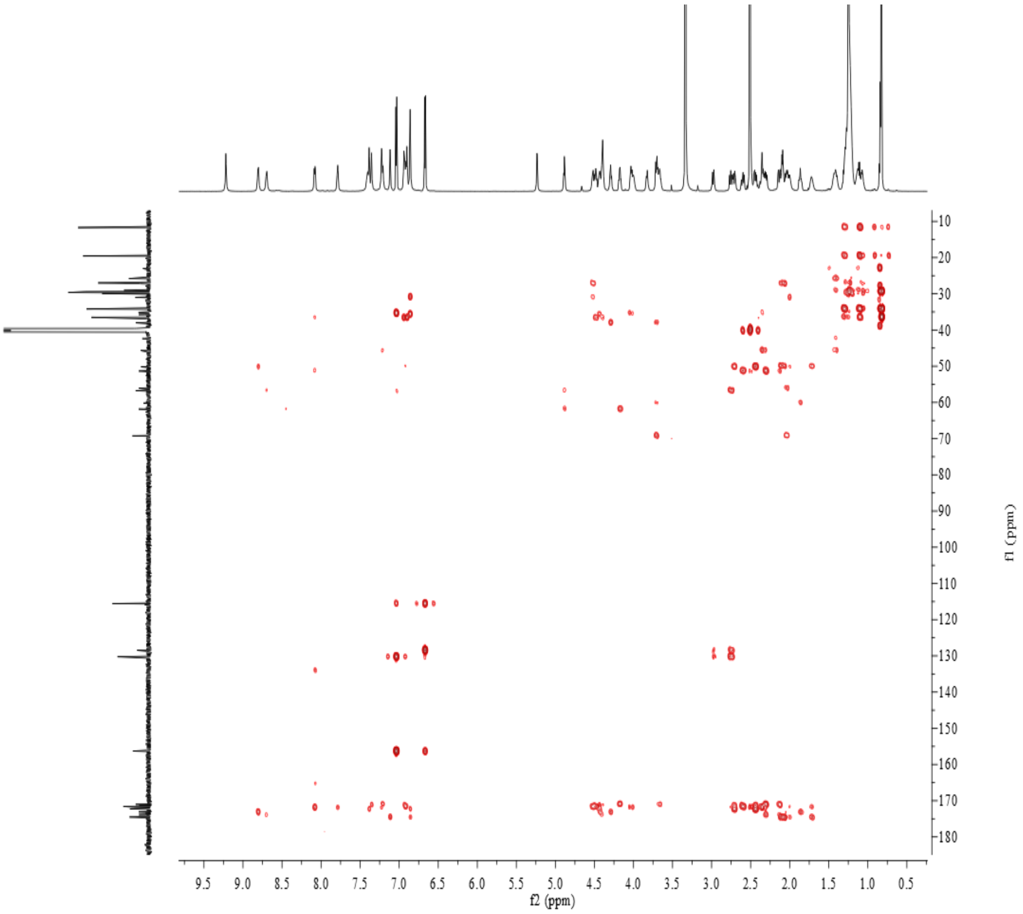

Figure S12. HMBC spectrum of **2**.

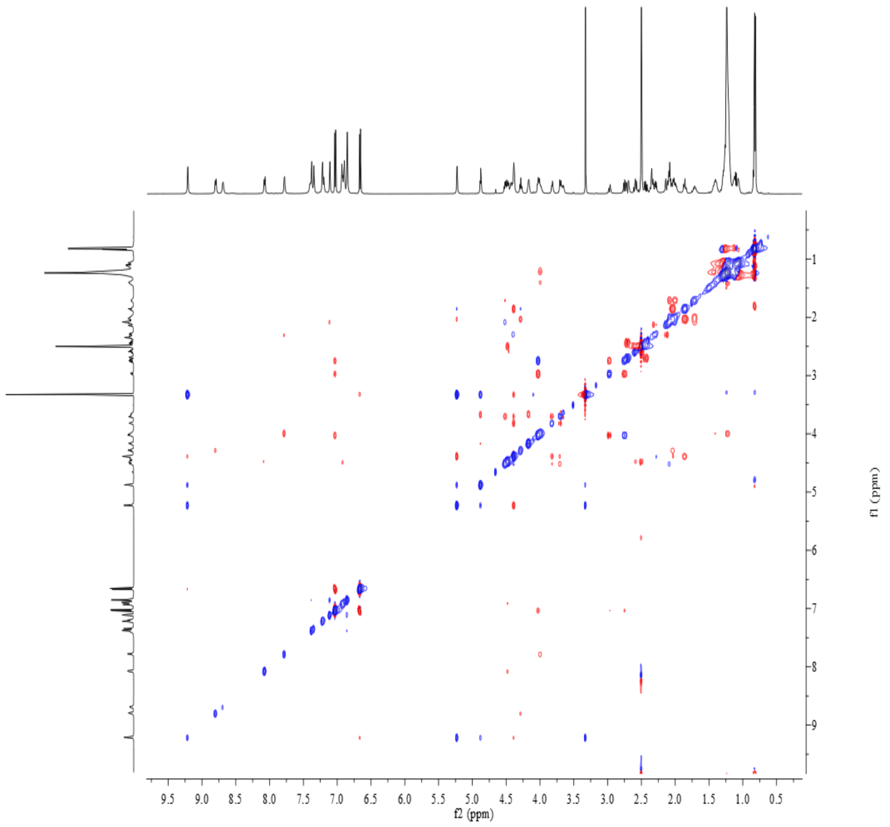

Figure S13. ROESY spectrum of **2**.

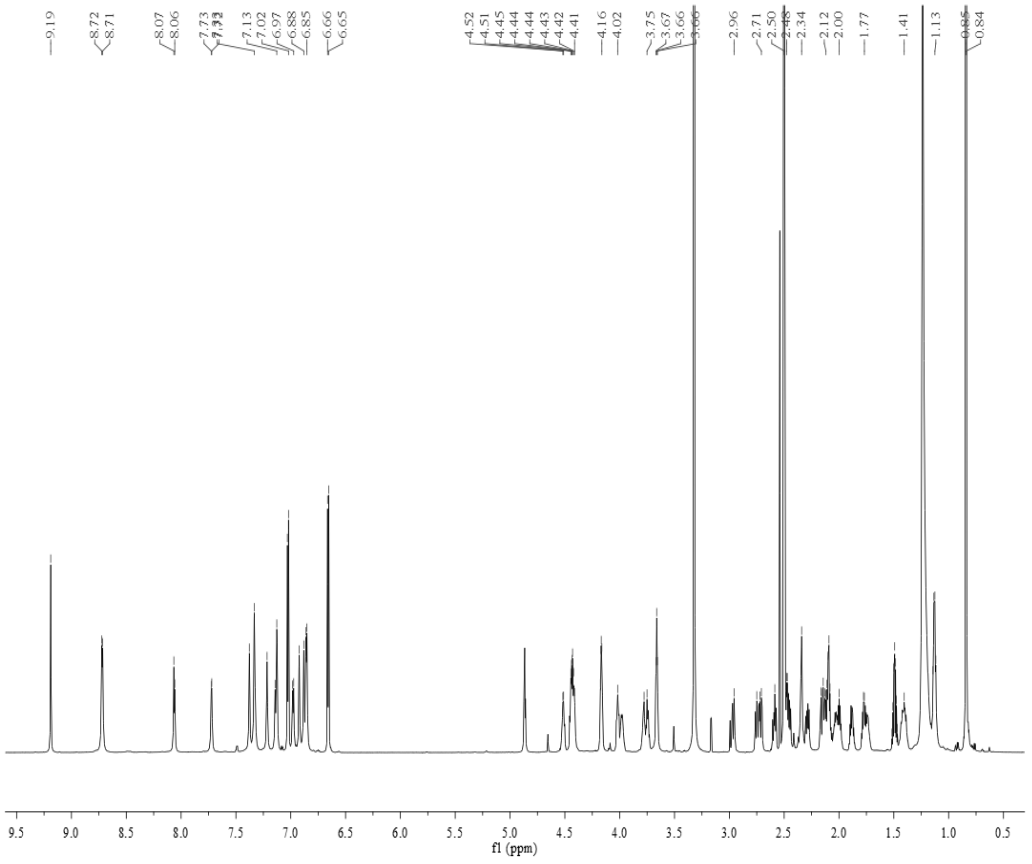

Figure S14. ^{1}H NMR (800 MHz, DMSO-*d6*) spectrum of **3**.

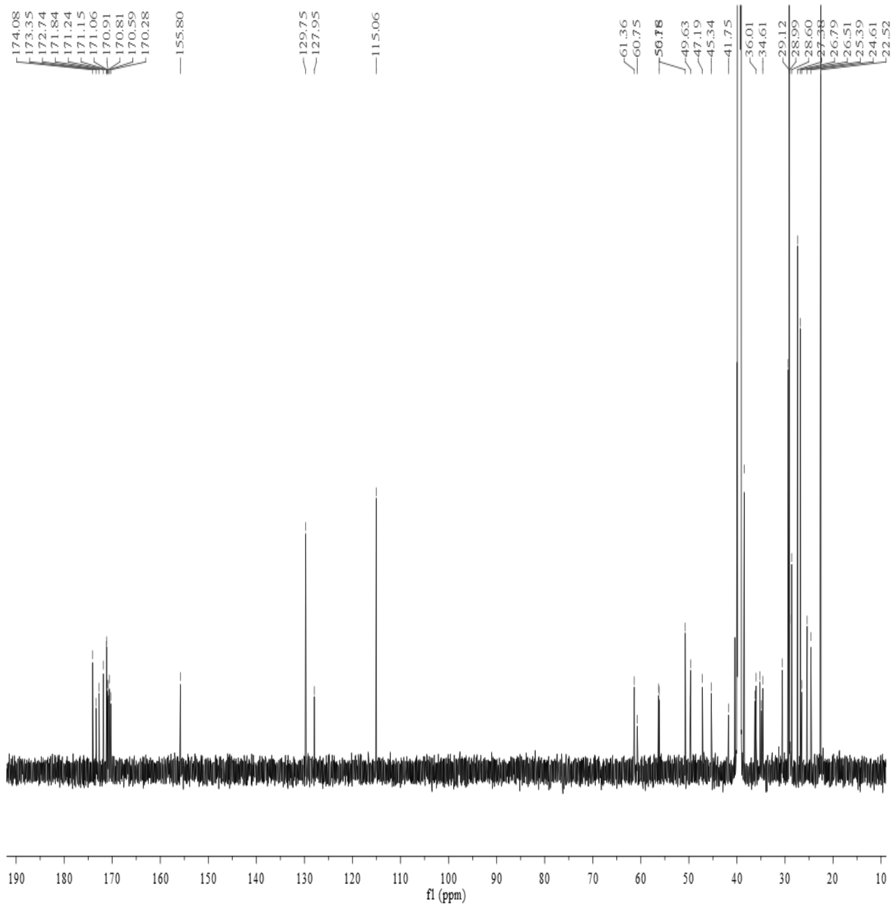

Figure S15. ^{13}C NMR (200 MHz, DMSO-*d6*) spectrum of **3**.

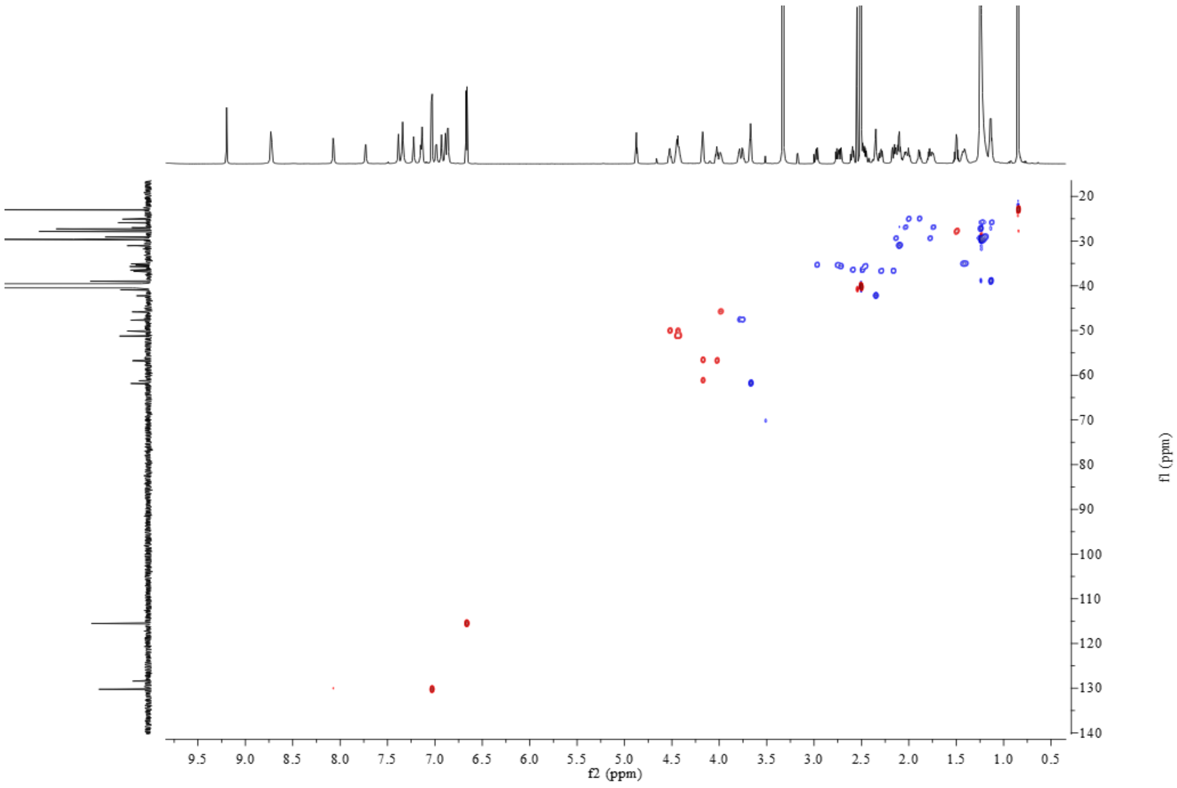

Figure S16. HSQC-DEPT spectrum of **3**.

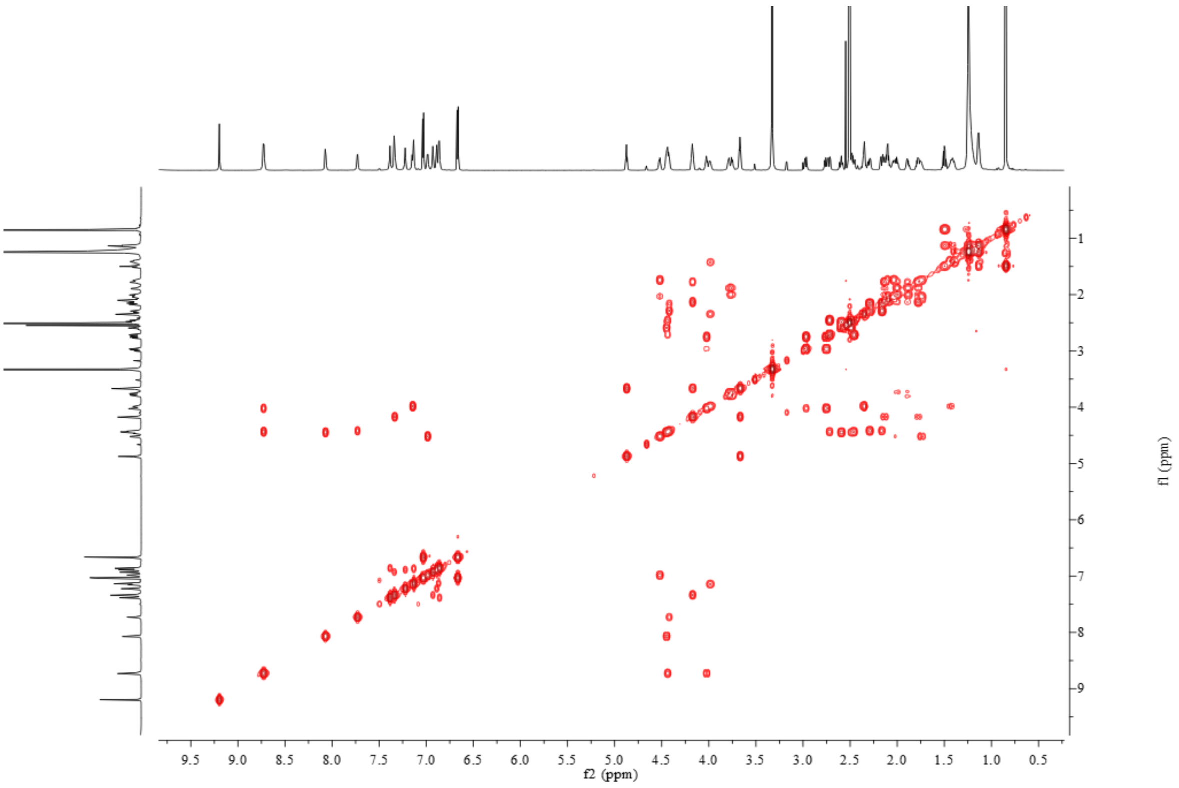

Figure S17. COSY spectrum of **3**.

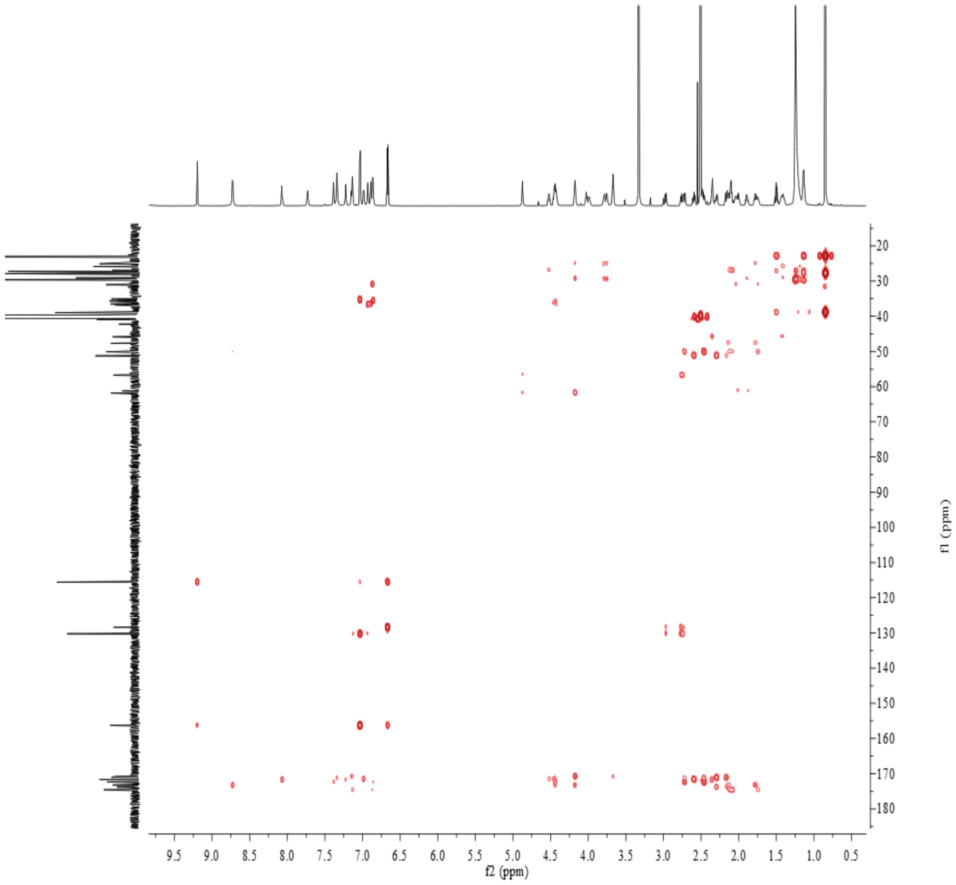

Figure S18. HMBC spectrum of **3**.

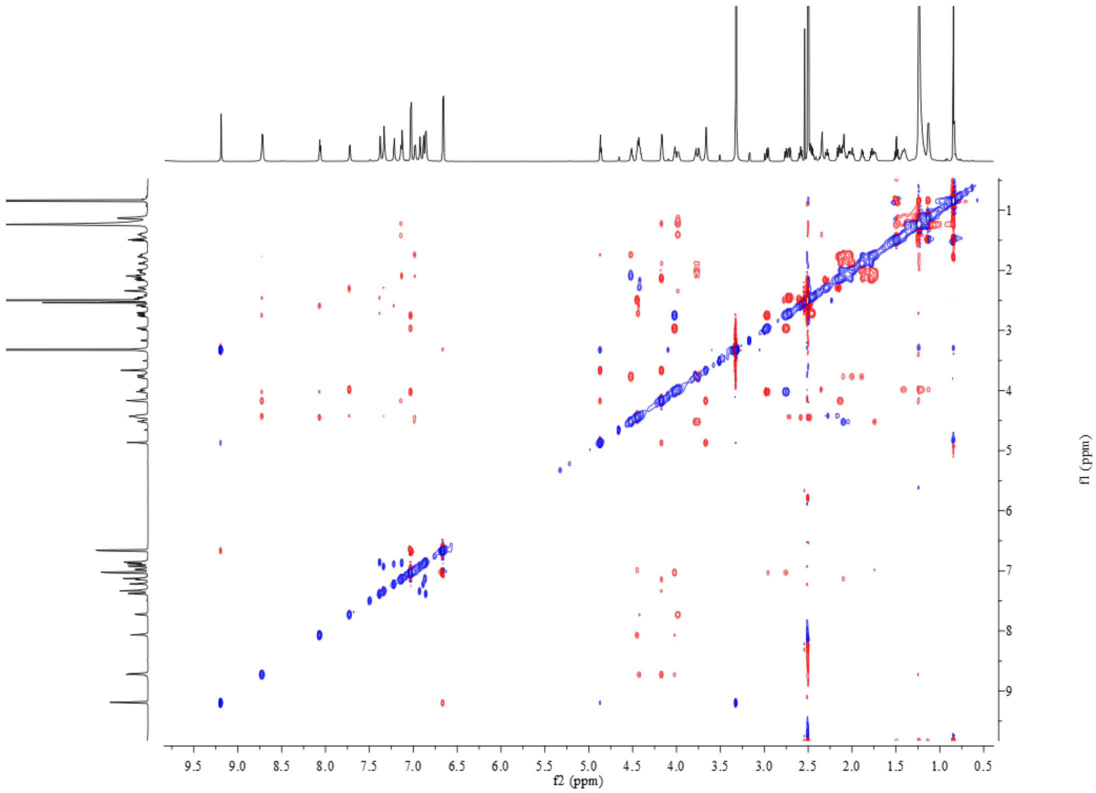

Figure S19. ROESY spectrum of **3**.

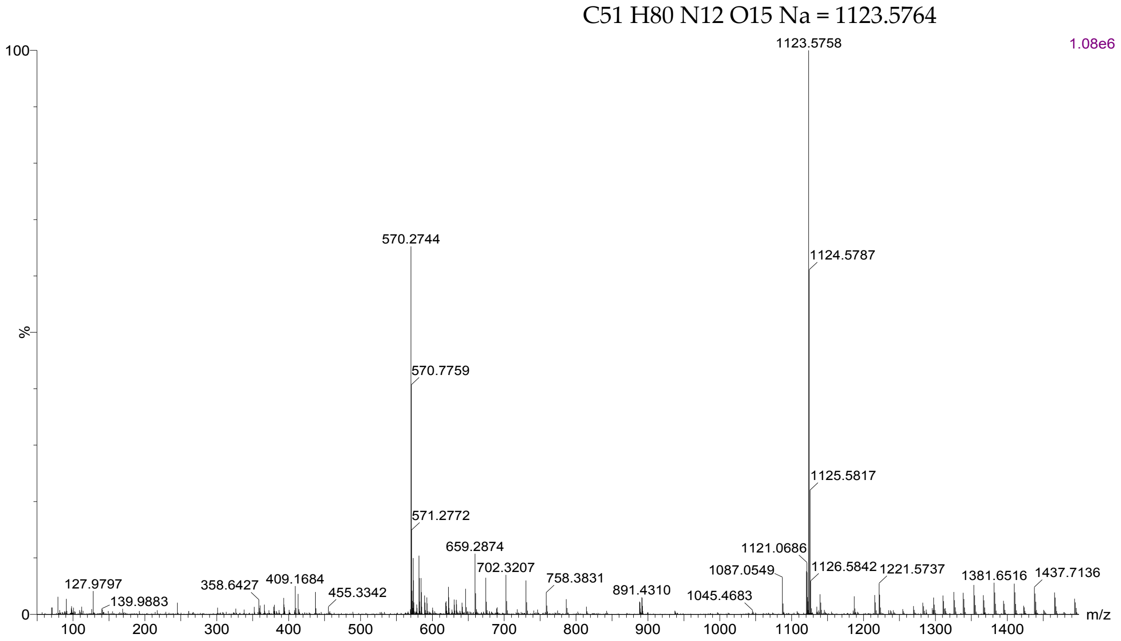

Figure S20. HRESIMS spectrum of **1**.

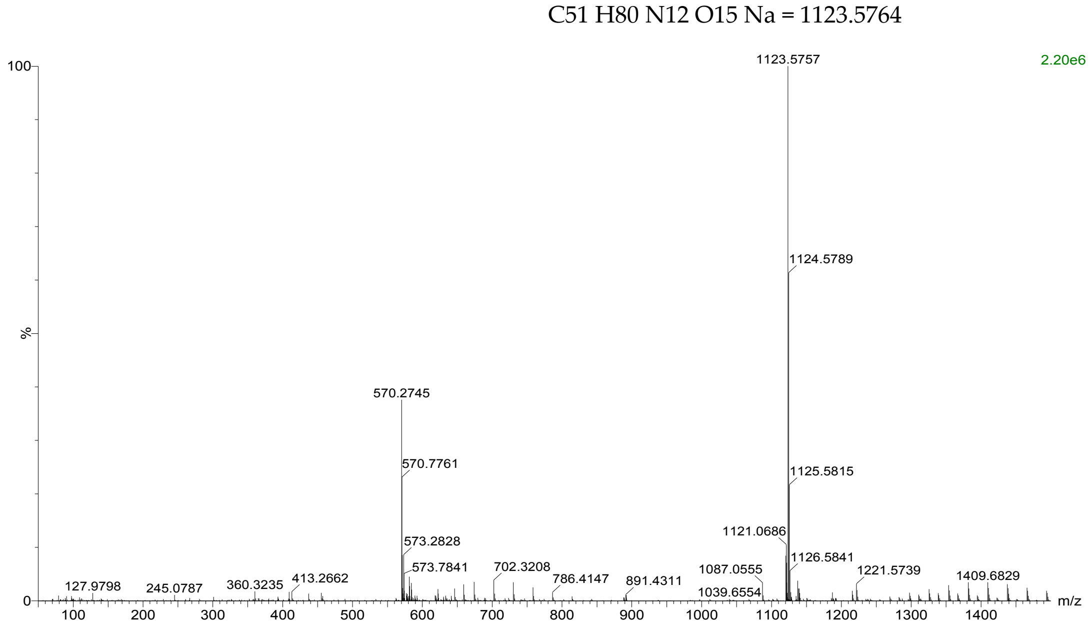

Figure S21. HRESIMS spectrum of **2**.

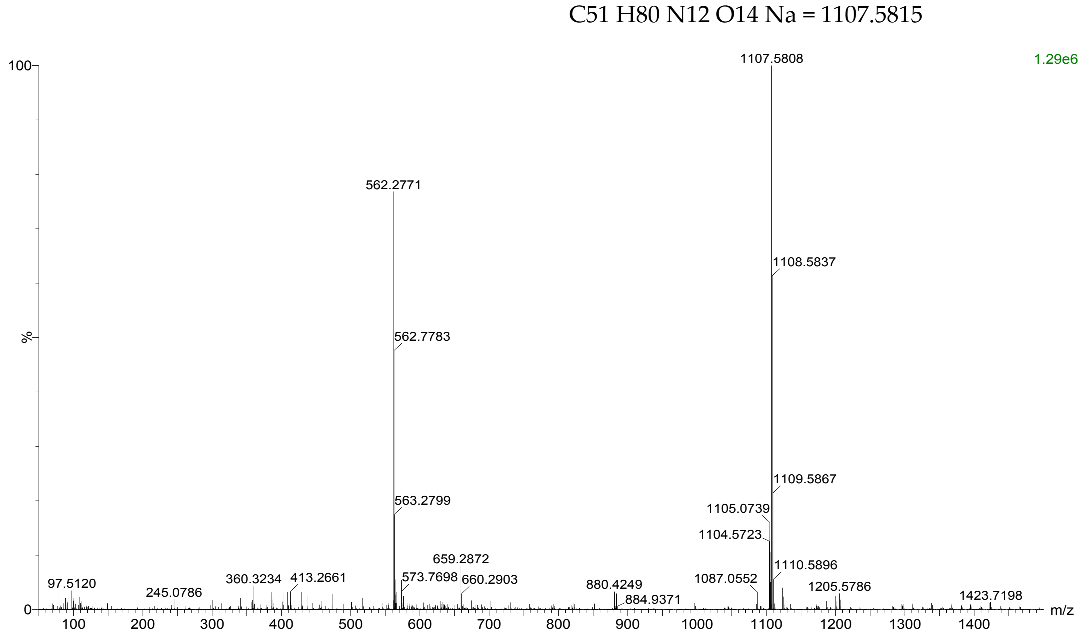

Figure S22. HRESIMS spectrum of **3**.

Table S1. Retention times (tR, min) of FDLA derivatives for **1–4**.

	**1**		**2**		**3**		**4**	
	t_{RL}	t_{RD}	t_{RL}	t_{RD}	t_{RL}	t_{RD}	t_{RL}	t_{RD}
Asn_1	11.04	11.20	11.02	11.18	11.04	11.20	11.04	11.20
Tyr	15.89	15.14	15.89	15.14	15.89	15.14	15.89	15.14
Asn_2	11.20	11.04	11.20	11.04	11.20	11.04	11.20	11.04
Gln	11.27	11.53	11.27	11.53	11.27	11.53	11.27	11.53
Pro	-	-	-	-	11.82	12.45	11.82	12.45
Asn_3	11.20	11.04	11.20	11.04	11.20	11.04	11.20	11.04
Ser	11.02	11.18	11.02	11.18	11.03	11.20	11.03	11.20

Table S2. Retention times (tR, min) of FDLA derivatives for 4-OH-Pro in 1 and 2, and standard amino acids.

	4-OH-Pro of **1**	4-OH-Pro of **2**	L-*trans*-4-OH-Pro	L-*cis*-4-OH-Pro	D-*trans*-4-OH-Pro	D-*cis*-4-OH-Pro
t_{RL}	10.32	10.32	10.31	10.59	10.34	10.71
t_{RD}	10.34	10.35	10.34	10.71	10.31	10.59

Table S3. Retention times (tR, min) of FDLA derivatives for β-amino fatty acids in **1–4**.

	**1**	**2**	**3**	**4**
t_{RL}	13.86	13.69	13.86	13.75
t_{RD}	8.69	8.75	8.67	8.55

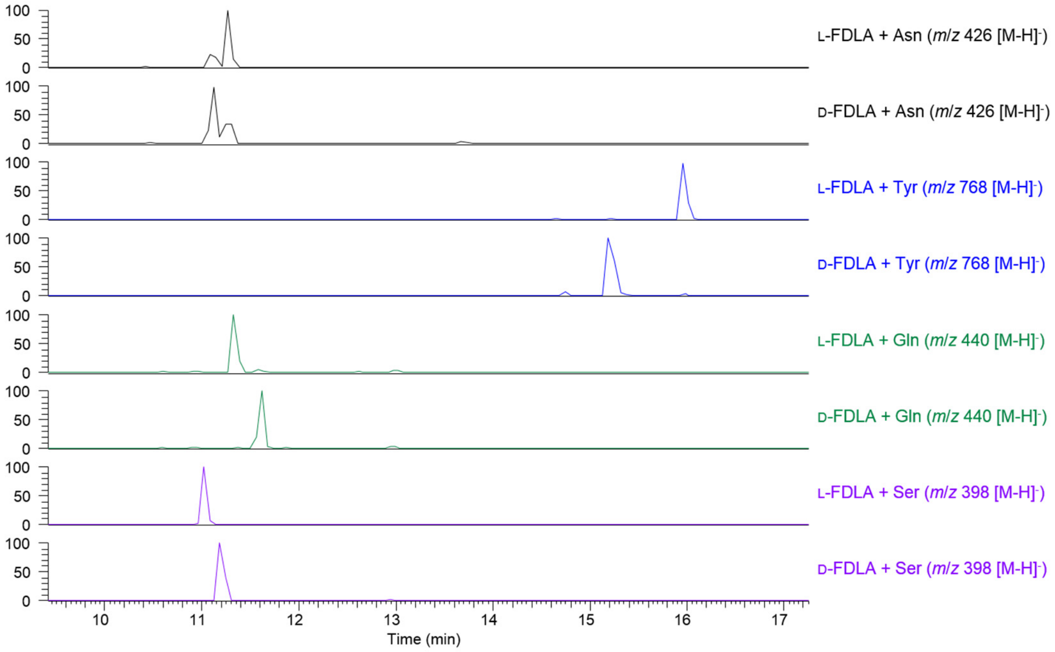

Figure S23. HPLC traces corresponding to Marfey's analysis of **1**.

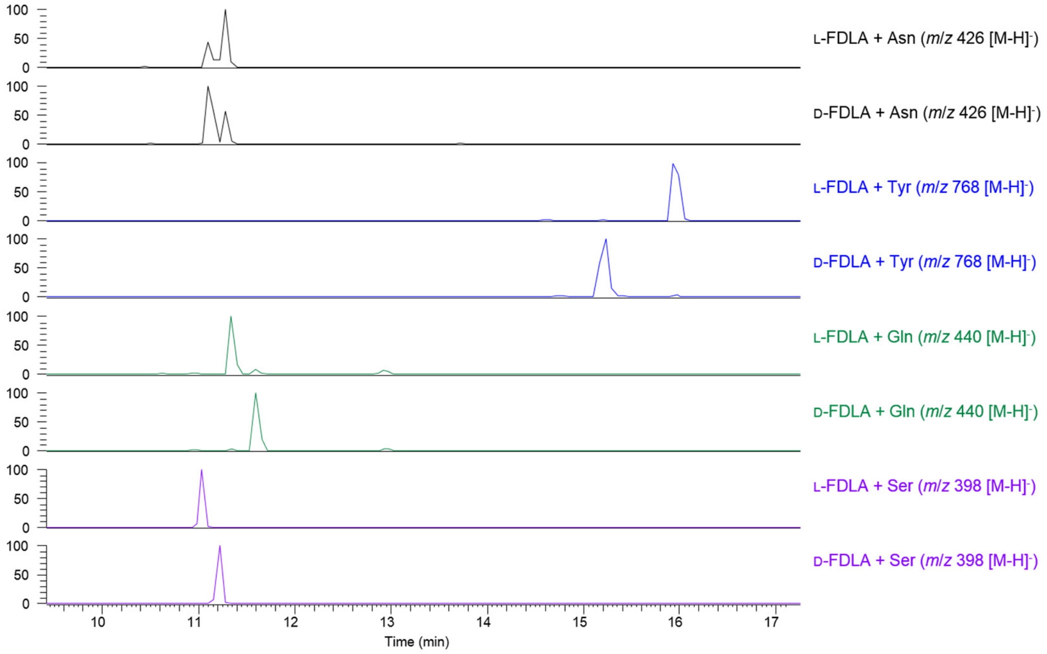

Figure S24. HPLC traces corresponding to Marfey's analysis of **2**.

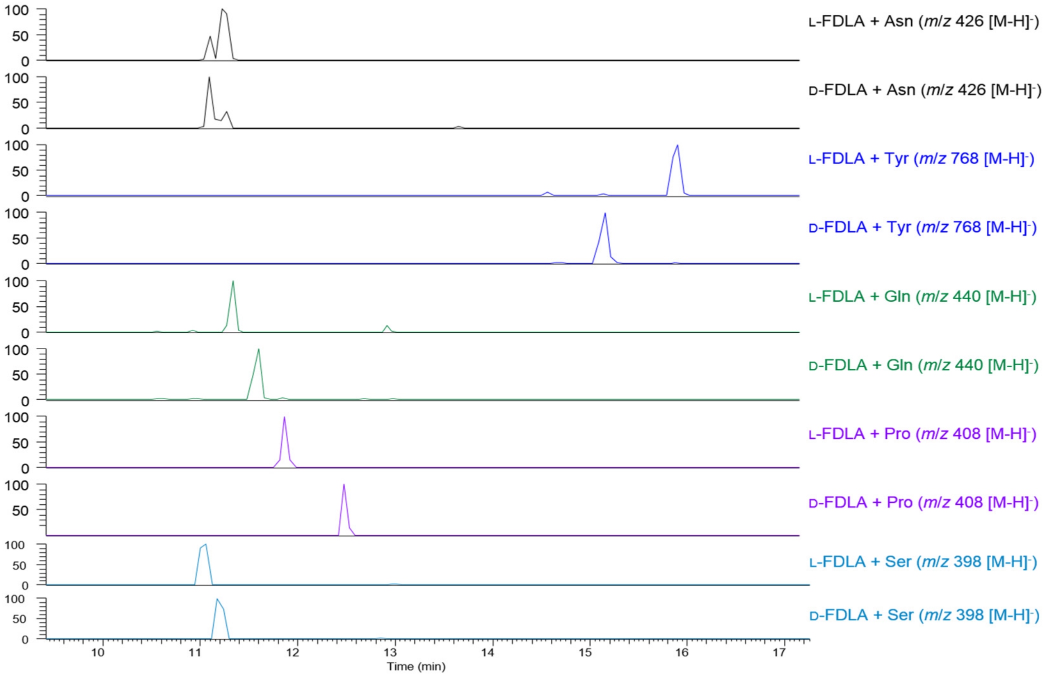

Figure S25. HPLC traces corresponding to Marfey's analysis of **3**.

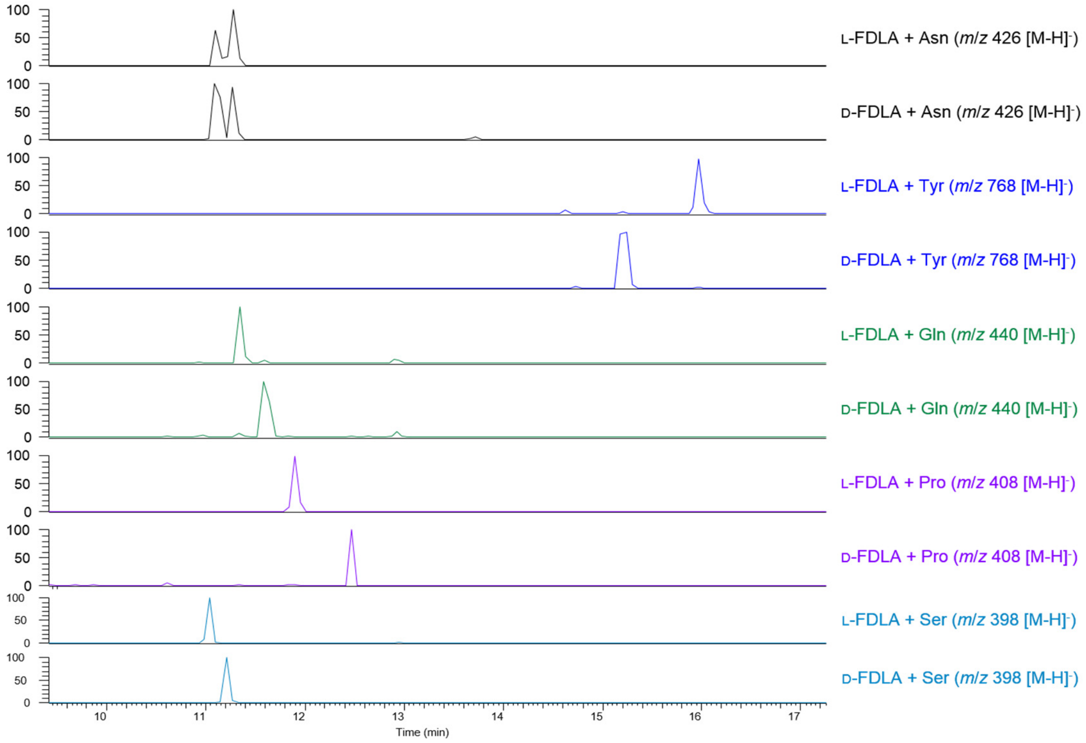

Figure S26. HPLC traces corresponding to Marfey's analysis of **4**.

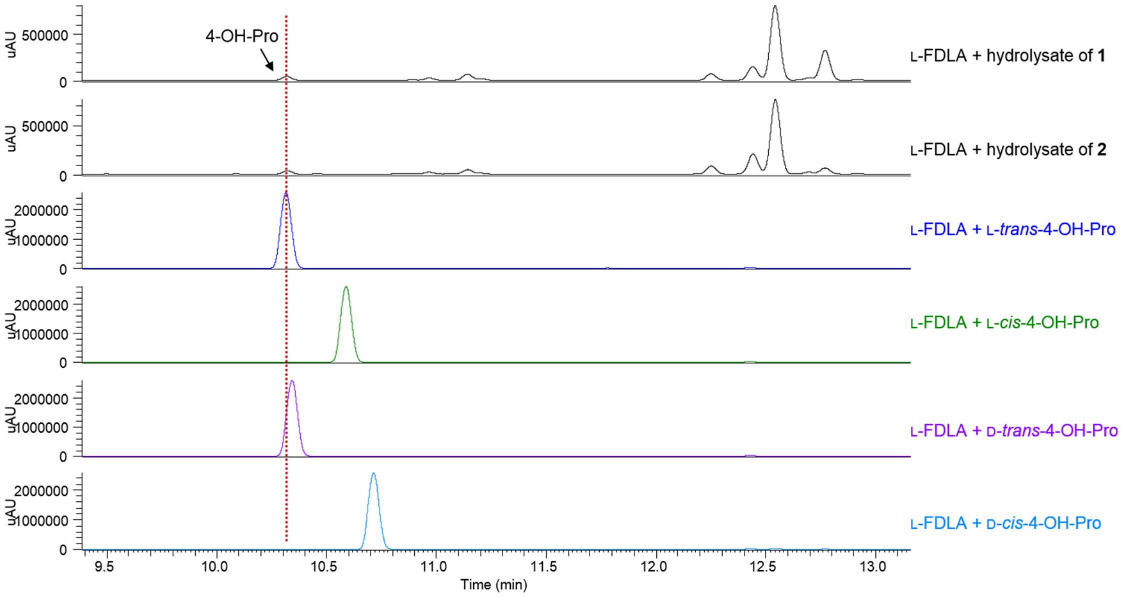

Figure S27. HPLC traces of L-FDLA derivatives of 4-OH-Pro in 1 and 2, and standard amino acids

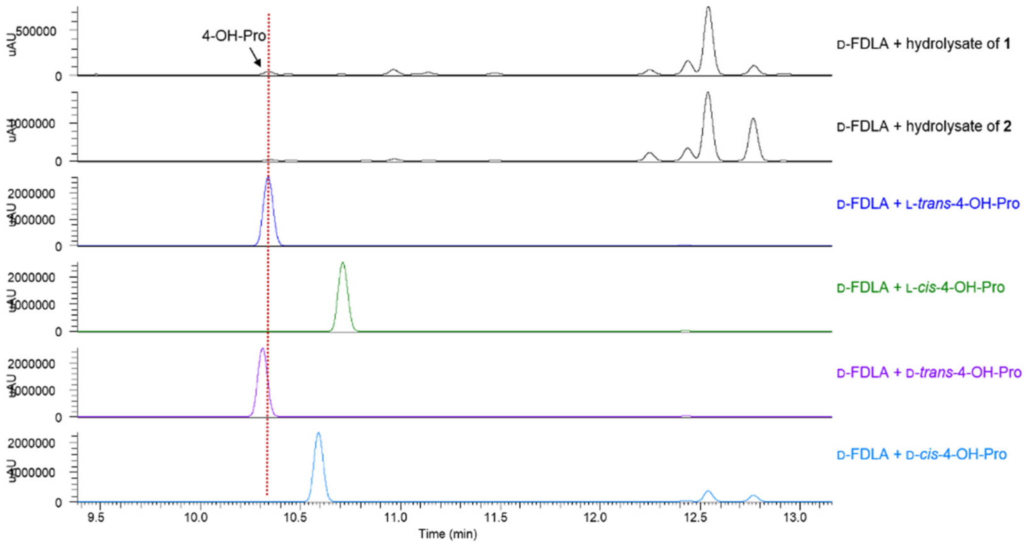

Figure S28. HPLC traces of D-FDLA derivatives of 4-OH-Pro in 1 and 2, and standard amino acids.

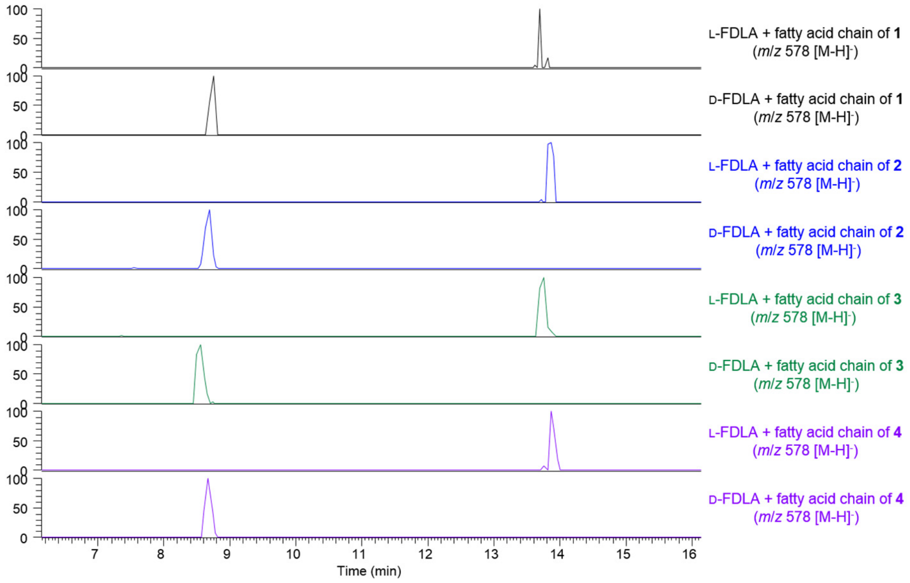

Figure S29. HPLC traces of L- and D-FDLA derivatives of fatty acid chains in 1–4.

(Solvent condition: 80–100% CH_3CN in H_2O over 15 min)
